# New insights from *Opisthorchis felineus* genome: update on genomics of the epidemiologically important liver flukes

**DOI:** 10.1186/s12864-019-5752-8

**Published:** 2019-05-22

**Authors:** Nikita I. Ershov, Viatcheslav A. Mordvinov, Egor B. Prokhortchouk, Mariya Y. Pakharukova, Konstantin V. Gunbin, Kirill Ustyantsev, Mikhail A. Genaev, Alexander G. Blinov, Alexander Mazur, Eugenia Boulygina, Svetlana Tsygankova, Ekaterina Khrameeva, Nikolay Chekanov, Guangyi Fan, An Xiao, He Zhang, Xun Xu, Huanming Yang, Victor Solovyev, Simon Ming-Yuen Lee, Xin Liu, Dmitry A. Afonnikov, Konstantin G. Skryabin

**Affiliations:** 1 Institute of Cytology and Genetics SB RAS, 10 Lavrentiev Ave, Novosibirsk, 630090 Russia; 2 Novosibirsk State University, 2 Pirogova Str, Novosibirsk, 630090 Russia; 3 Russian Federal Research Center for Biotechnology, 33/2 Leninsky prospect, Moscow, 119071 Russia; 4 BGI-Shenzhen, 11 Beishan Industrial Zone, Yantian District, Shenzhen, 518083 China; 5 State Key Laboratory of Quality Research in Chinese Medicine, Institute of Chinese Medical Sciences, University of Macau, Macao, China; 6 Federal Research Center Kurchatov Institute, Moscow, Russia; 7 ZAO Genoanalytica, 1 Leninskie Gory street, Moscow, 119234 Russia; 8 Softberry Inc., 116 Radio Circle, Suite 400, Mount Kisco, NY 10549 USA

**Keywords:** Opisthorchiidae, *Opisthorchis felineus*, Genome, Trans-splicing, Microintrons, Liver flukes, Transcriptome, Metacercariae

## Abstract

**Background:**

The three epidemiologically important Opisthorchiidae liver flukes *Opisthorchis felineus*, *O. viverrini*, and *Clonorchis sinensis*, are believed to harbour similar potencies to provoke hepatobiliary diseases in their definitive hosts, although their populations have substantially different ecogeographical aspects including habitat, preferred hosts, population structure. Lack of *O. felineus* genomic data is an obstacle to the development of comparative molecular biological approaches necessary to obtain new knowledge about the biology of Opisthorchiidae trematodes, to identify essential pathways linked to parasite-host interaction, to predict genes that contribute to liver fluke pathogenesis and for the effective prevention and control of the disease.

**Results:**

Here we present the first draft genome assembly of *O. felineus* and its gene repertoire accompanied by a comparative analysis with that of *O. viverrini* and *Clonorchis sinensis*. We observed both noticeably high heterozygosity of the sequenced individual and substantial genetic diversity in a pooled sample. This indicates that potency of *O. felineus* population for rapid adaptive response to control and preventive measures of opisthorchiasis is higher than in *O. viverrini* and *C. sinensis*. We also have found that all three species are characterized by more intensive involvement of trans-splicing in RNA processing compared to other trematodes.

**Conclusion:**

All revealed peculiarities of structural organization of genomes are of extreme importance for a proper description of genes and their products in these parasitic species. This should be taken into account both in academic and applied research of epidemiologically important liver flukes. Further comparative genomics studies of liver flukes and non-carcinogenic flatworms allow for generation of well-grounded hypotheses on the mechanisms underlying development of cholangiocarcinoma associated with opisthorchiasis and clonorchiasis as well as species-specific mechanisms of these diseases.

**Electronic supplementary material:**

The online version of this article (10.1186/s12864-019-5752-8) contains supplementary material, which is available to authorized users.

## Background

*Opisthorchis felineus* (Rivolta, 1884) is a member of the triad of epidemiologically important fish-borne liver trematodes, which also includes *O. viverrini* (Poirier, 1886) and *Clonorchis sinensis* (Loos, 1907). These liver flukes are known to cause serious human diseases affecting bile ducts and the gall bladder. Liver fluke infection is recognized as the major risk factor of cholangiocarcinoma [1–3]. An estimated 12.5, 67.3 and 601 million people are currently at risk for infection with *O. felineus*, *O. viverrini* and *C. sinensis*, respectively [4]. According to the Food and Agriculture Organization and World Health Organization [5, 6], these liver flukes are the 8th in the overall global list of 24 food-borne parasites.

**Fig. 1:**
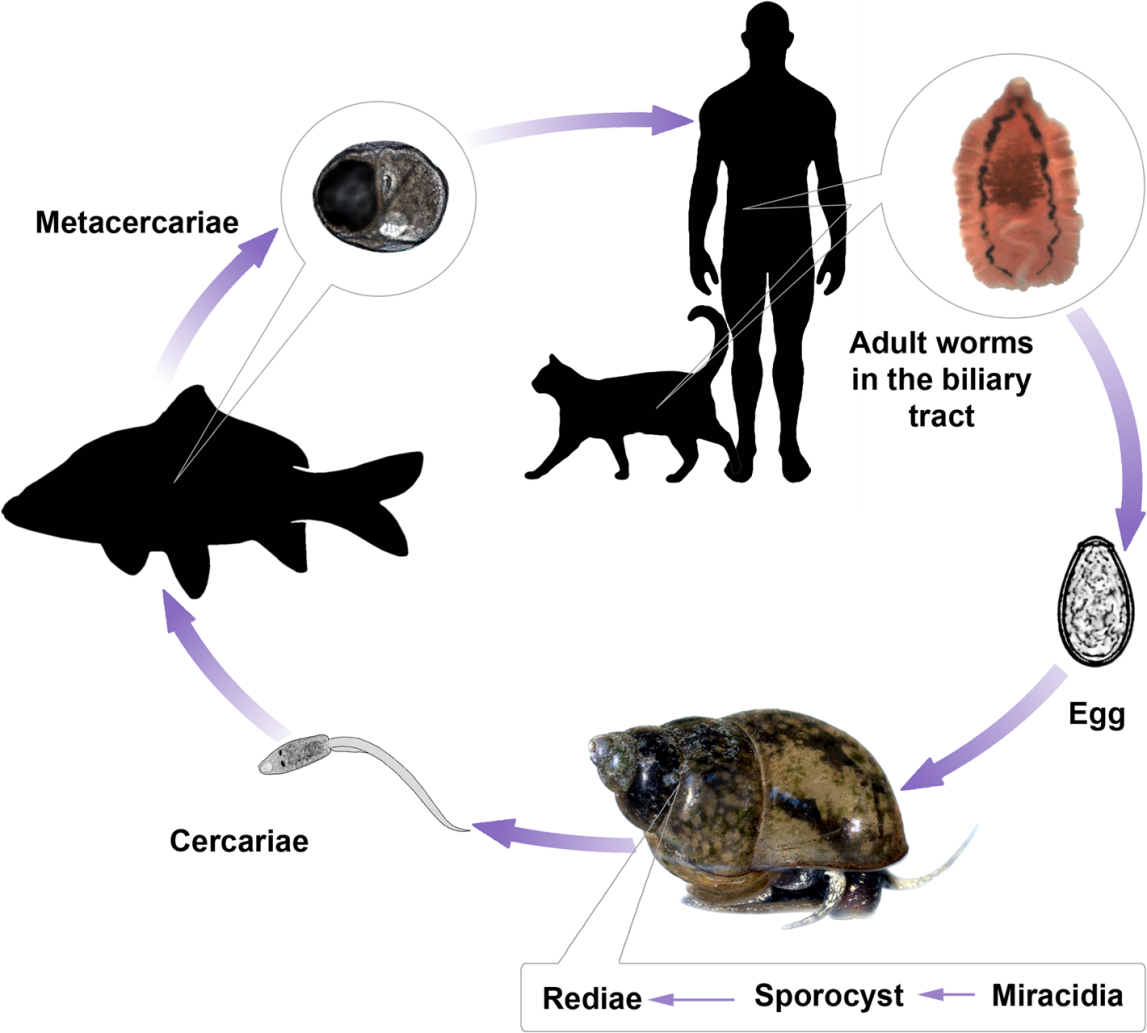
Life cycle of *Opisthorchis felineus*. The eggs are shed in the biliary tree of fish-eating mammals and are passed with feces. They need to be ingested by freshwater gastropod snail, the first intermediate host, to develop into sporocysts, rediae, and free-swimming cercariae, the stage infective for the second intermediate host, cyprinoid fish [3, 7]. Humans and other fish-eating mammals may serve as a definitive host by ingesting raw, slightly salted, or frozen fish. Entering the host body, metacercariae infect the biliary tract of mammals, where they mature into adult worms, the sexual stage, over approximately 1 month. The lifespan of an adult liver fluke in the human body can reach over 20 years [7]

The liver flukes *O. felineus, O. viverrini,* and *C. sinensis* are typical trematodes with an intricate life cycle including alternation of two intermediate hosts and one definitive host (Fig. 1). Liver flukes are capable of infecting humans, wild and domestic fish-eating animals. The definitive hosts are infected by ingesting a raw or undercooked fish containing metacercariae. Once the metacercaria enters the digestive tract, its envelope is destroyed and the excysted liver fluke penetrates the hepatobiliary system of fish-eating mammals [3, 7, 8].

The three epidemiologically important liver fluke species have distinct differences in the geography and origin of their foci. Endemic area of *O. felineus* far exceeds areas of *O. viverrini* and *C. sinensis* and extends to several climatic zones - from the Arctic Circle in Western Siberia to Southern Europe [3]. The world’s largest center of opisthorchiasis felinea is located in the basin of the rivers Ob and Irtysh. Fish contamination in the north of this region, where the population density is 1–2 inhabitants per square km, exceeds 90% [3]. This indicates that the main reservoir of the *O. felineus* in the Ob-Irtysh basin are wild animals. European foci of opisthorchiasis felinea are also likely supported by feral carnivores, since human cases are quite rare [9].

Distribution area for *O. viverrini* is Southeast Asia (Thailand, Lao PDR, Cambodia and central to southern Vietnam). It is believed that zoonotic cycle has largely disappeared for this fluke, being replaced with a predominantly anthropogenic cycle [9]. *C. sinensis* is endemic to East Asia (China, Korea, Russian Far East and Japan) and northern Vietnam [4, 10] and holds an intermediate position, with a number of significant native and domestic reservoir animal hosts but also with high levels of human fecal contamination of the environment playing a significant role in the transmission cycle [10]. Correspondingly, *O. felineus, O. viverrini*, and *C. sinensis* also display certain differences in the range of their primary and secondary hosts [7–10].

Epidemiologically important liver flukes differ also in population structure. Analysis of mitochondrial and nuclear genetic markers revealed that population structure is absent in *O. felineus* across Eastern Europe, Northern Asia (Siberia) and Central Asia (Northern Kazakhstan) [11]. In contrast, population genetic differentiation exists in *O. viverrini* [12]. Genetic diversity of *C. sinensis* is not as pronounced as it is for *O. viverrini*, nevertheless geographic variation in *C. sinensis* was detected [13]. Recently it has been shown that *O. viverrini* differs from *O. felineus* and *C. sinensis* in chromosome number. Karyotypes of *O. felineus* and *C. sinensis* (Russian isolate) consist of two pairs of largemeta- and submetacentrics and five pairs of small chromosomes (2n = 14). However, the karyotype of *O. viverrini* is 2n = 12 [14].

Thus, these liver flukes are attractive research objects from the standpoint of comparative genomics allowing for better insight into the mechanisms underlying the evolution and adaptation of trematodes. Taking into account the importance of opisthorchiasis and clonorchiasis for the population health in endemic regions, the genomic studies of these infectious agents can give a clue to solving many applied problems and are a priority direction in the modern molecular biology.

*O. viverrini* and *C. sinensis* but not *O. felineus* have been recently characterized at the level of genome [15–17]. The results have significantly enriched our understanding of the molecular processes that ensure the vital activity of these parasites in the bile duct, and expanded the knowledge about liver fluke-associated carcinogenesis. However, the genomics of O. felineus is poorly investigated and this hinders a deep understanding of the biology of this parasite and the progress in comparative genomics of opistorchiids. To address this knowledge gap, we have sequenced the *O. felineus* genome and used the de novo assembled draft genome to gain new insights into genetic features of the liver flukes. Here we present the first version of *O. felineus* draft genome assembly and the accompanying transcriptome assembly. We also provide *O. felineus* genome annotation and describe the results of the first comparative analysis of *O. felineus*, *O. viverrini* and *C. sinensis* genomics and transcriptomics, including taxa-specific features of RNA processing. Although the coding regions of the genes are highly homologous to each other; however, analysis of the genome-wide synteny between *O. felineus*, *O. viverrini* and *C. sinensis* demonstrates a considerable variation in the liver fluke genomes. The majority of genes in adult worms demonstrate similar level of mRNA expression among these species. We also found that trans-splicing potentially plays an important role in RNA processing of these three liver flukes.

## Results

### Genome assembly revealed high heterozygosity rate

**Fig. 2:**
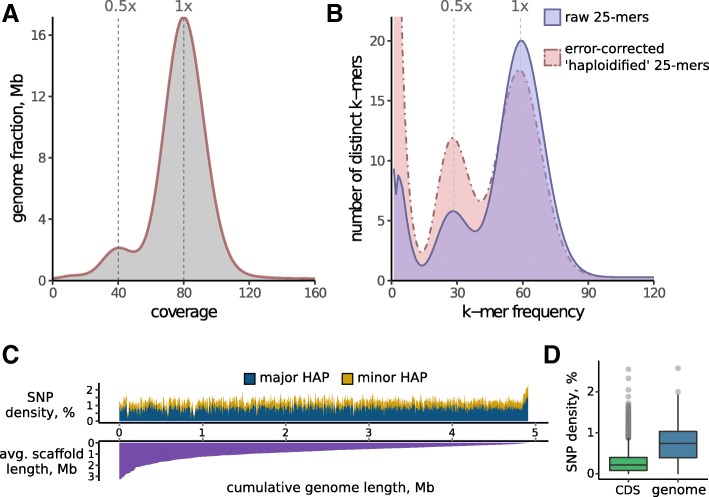
Genetic variation in the draft genome assembly of *O. felineus*. Genome coverage (**a**) and K-mer frequency distribution (**b**) by paired-end library prepared from single-worm sample are presented. The plots demonstrate high heterozygosity level of the sample. Major peak (homozygous genome fraction, k-mers common to both haplotypes) is supplemented with the minor one at twice lower coverage corresponding to the k-mers associated with polymorphisms. **c**. Distribution of SNP density throughout the genome. In addition to the two major haplotypes of sequenced individual, two minor haplotypes (up to 9% each), possibly originating from the genetic material of a sexual partner, were also observed. Genomic scaffolds were arranged by their length as shown in the bottom panel, and densities were calculated in 500-kb windows. **d**. Boxplot of SNP density in genomic and coding regions

Assembly of the genomes of pooled samples collected from native populations is often hampered by high levels of genetic variation (heterozygosity), resulting in excessively large and highly fragmented draft genomes. To avoid this, we performed deep genome sequencing of a single worm, customizing the design of short-insert libraries to the requirements of Allpaths-LG assembler (Additional file 2: Table S1). For efficient scaffolding, several long-insert libraries (Additional file 2: Table S1) prepared from pooled samples were also sequenced, totaling ~ 40 Gb of the data. The data were sufficient to produce a 684 Mbp genome assembly with an acceptable N50 value of 624 Kb (Table 1) and relatively low sequence redundancy, as evidenced from the distribution of coverage by genomic libraries (Fig. 2a). The *O. felineus* genome size was slightly longer as compared with *С. sinensis* (547 Mb) and almost the same as the *O. viverrini* genome (634.5 Mb) [16, 17]. The GC-content of the resulting genome appeared to be very close to those of *С. sinensis* and *O. viverrini*. In total, 11,455 protein-encoding genes (Table 1) were predicted from the genome based on transcriptomic evidence from previously published [18] and new (Additional file 2: Table S1) RNA-seq data and sequence similarity to protein-encoding genes of *C. sinensis* and *O. viverrini*. The estimated total number of genes, the proportion of coding regions (2.8%), the mean total gene length (25,615 bp), intron length (3546 bp) and the mean number of exons per gene [9] were similar to those of *C. sinensis* and *O. viverrini* [16].

**Table 1:** Characteristics of the *Opisthorchis felineus* draft genome assembly

Characteristics of the genome assembly	
Total size of scaffolds (bp)	683,967,183
Number of scaffolds	13,781
Longest scaffold (bp)	3,238,362
Number of scaffolds: > 1 kb; > 50 kb	13,511; 1489
N50/N75 scaffold length (bp)	624,179/309,294
Genomic DNA GC content (excluding Ns)	44.07%
Draft genome features^a^	
Length of CDS domain in the genome	19,274,911
Predicted genes	11,455
Predicted protein-coding mRNA sequences	21,036
Gene average length	25,615
Coding domain length	1732
Average number of exons	9
Average length of exons	1908
Average length of introns	3546

^a^Statistics of the gene annotation using EVidenceModeler prediction approach is presented. The full data of statistics of the gene annotations produced by several prediction approaches is presented in Additional file 2: Table S5

One of the main factors that hampered the contiguity of the assembly produced was high heterozygosity of the sequenced single-worm sample. According to the analysis of genome sequence and coverage, the observed sequence heterogeneity is not attributed to mitochondrial DNA or contamination by host or bacterial DNA. In the k-mer frequency distribution analysis of paired-end library performed as part of Allpaths-LG pipeline, the heterozygous fraction of the genome generated an additional minor peak with a maximum at twice lower frequency compared to the main peak; the former was efficiently collapsed up to the major frequency by Allpaths-LG ‘haploidify’ algorithm (Fig. 2b). The het-rate inferred from the distribution of raw k-mers was nearly 1 per 100 bp. We have further explored putatively high *O. felineus* genome variation by variant calling from the genome-mapped data, excluding the loci with strongly biased coverage (Fig. 2a). In addition to the two major haplotypes corresponding to the diploid set of sequenced individual, two minor haplotypes (up to 9% each), possibly originating from the genetic material of a sexual partner, were also observed throughout the genome (Fig. 2c). After strict filtering of these low-frequency SNPs, the resulting heterozygosity rate of the assembled individual still remained high, being 1/131 bp for the whole genome and 1/357 bp for its protein-coding fraction (Fig. 2d).

### Repetitive elements, genome-wide synteny and phylogenetic relationships

We used the assembled *O. felineus* genome together with the available genomes of *O. viverrini, C. sinensis,* and *F. hepatica* (as an outgroup) in a comparative analysis of the content and dynamics of repetitive sequences in the liver fluke genomes.

Two separate libraries of genomic repeats were constructed for the three liver fluke species and *F. hepatica* using both the known (RepBase) and de novo predicted (Tedna and RepeatModeler) repetitive elements. The analysis showed an extremely low overlap between the Tedna and RepeatModeler repeat libraries (< 0.1%) for each of the genomes, demonstrating the advantage of using both methods. The repeats accounted for 30.3, 30.9, 29.6, and 55.3% of *O. felineus*, *O. viverrini*, *C. sinensis*, and *F. hepatica* genomes (Additional file 2: Tables S3.1–3.4), respectively. The total numbers obtained for the *O. viverrini* and *C. sinensis* genomes are consistent with those obtained in previous studies (30.6% for *O. viverrini* [16] and 32% for *C. sinensis* [17], but with a larger share of annotated elements (9.3 and 14.3%, respectively) and different ratios of repeat superfamilies (Additional file 2: Table S3.3–3.4). While the *F. hepatica* genome was earlier reported to be 32% repetitive [19], we found at least 54% of the genome masked for annotated transposons only, not taking into account the tandem, satellite, and other simple repeats. This feature of *F. hepatica* genome can partly explain its considerably larger size as compared with other studied trematodes (Additional file 2: Table S3.1).

The majority of repeats (90.2%) in the *O. felineus* genome are retrotransposons, with 17.9% of LTR, 72.3% of LINE, and 0.4% of SINE elements, while the remaining 9.8% are formed by cut-and-paste DNA transposons (Additional file 2: Table S3.2). The overall repeat landscape of *O. felineus* genome is similar to those of the other three trematodes in question (Additional file 2: Table S3.1-ST3.4; Additional file 1: Figure S4). Divergence of transposable element copies from their consensus is correlated with the age of their activity. More similar copies (low distance from the consensus) are indicative of recent activity of an element and vice versa [20]. We found that the majority of the transposable element copies identified in the studied opisthorchiid genomes have a similar distance from their corresponding consensuses (approximately 20%) (Additional file 1: Figure S4), indicating the same time of the last transposition burst in these genomes.

We conducted genome-wide synteny comparisons between *O. felineus*, *O. viverrini, C. sinensis,* and *Schistosoma mansoni*. The genomic sequences of these flukes were compared in a pairwise manner using MUMmer (see Methods). The large (> 100 kb) scaffolds of *O. felineus, O. viverrini,* and *C. sinensis* at the level of amino acid sequences display nearly the same level of differences as compared to the first chromosome of *S. mansoni* genome. The amino acid identity and similarity in the alignment was approximately 70 and 80%, respectively (Additional file 2: Table S4). The identity and similarity parameters for *O. felineus* are somewhat higher as compared with the other two opisthorchiids.

A comparison of three pairs of liver fluke genomes at a nucleotide level without filtering repeats demonstrates that the pair *O. felineus*–*C. sinensis* has the highest similarity as compared with the remaining two pairs (Additional file 2: Table S4). The largest number of aligned scaffolds for both the reference (*O. felineus*) and query (*C. sinensis*) has been detected for this genome pair as well as a large share of aligned nucleotides, accounting for 40 to 50% of the total length of the reference and query, respectively. In addition, the average length of aligned fragments for this pair is the longest (~ 1600 bp versus ~ 1200 bp for the remaining pairs) and the alignments display a higher level of nucleotide identity (84.2 versus 83.5%). The comparison of the *O. viverrini* and *C. sinensis* both to each other and to *S. mansoni* chromosome 1 fits well the data obtained by Young et al. [16].

Similar results were obtained when comparing the liver fluke genomes at the level of amino acid sequences (Additional file 2: Table S4). Comparison of the amino acid sequence of the pair *O. felineus*–*C. sinensis* shows the largest number of aligned scaffolds, longest homologous regions, and highest sequence similarity as compared with the other genome pairs. Analysis of the genomic sequences with masked repeats suggests an analogous inference as well as analysis of the characteristics of syntenic blocks in the three genomes by the SyMap software [21] using both unmasked and masked sequences (Additional file 2: Table S4).

We additionally analyzed synteny between three Opistorchiidae genomic sequences using OrthoCluster software (see Methods). Results demonstrate (Additional file 2: Table S4) that the synteny conservation between *O. felineus* and *C. sinensis* is higher (0.363) than that for *O. viverrini* and *C. sinensis* (0.215) or *O. felineus* and *O. viverrini* (0.256). Other parameters of OrthoCluster syntenic block comparison demonstrates also that the genomic structure *O. felineus* and *C. sinensis* is more similar (Additional file 2: Table S4). This is in accordance with genomic sequence alignment results (Additional file 1: Figure S3).

**Fig. 3:**
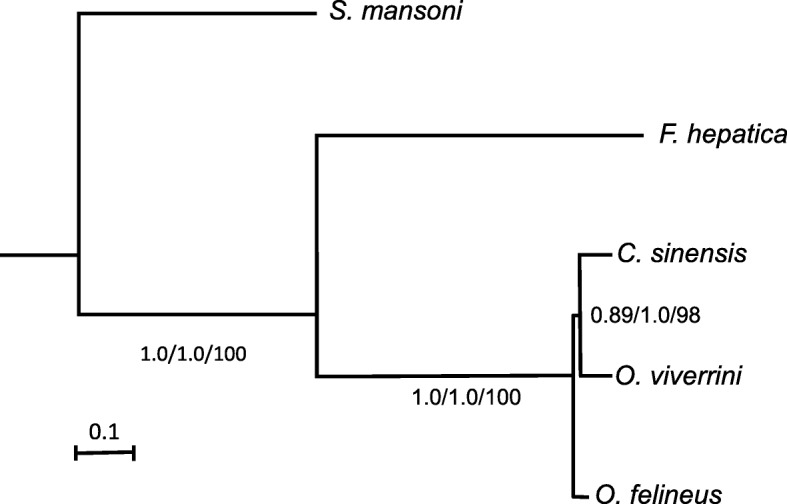
Phylogenetic trees reconstructed according to aligned amino acid sequences of the *S. mansoni*, *Fasciola hepatica*, *O. felineus*, *C. sinensis*, and *O. viverrini* genomes using MrBayes software. The topology of the trees obtained by Phylobayes and RAxML is the same. The scale of the evolutionary distance is shown below. The node support/bootstrap values are shown for MrBayes/Phylobayes/RAxML, respectively

The phylogenetic relationships between three liver fluke species were reconstructed using three methods; in general, topologies of the resulting trees were similar (Fig. 3). All the trees coincided in their topologies and were very similar in the ratios of branch lengths and node support (bootstrap values); correspondingly, only the tree generated by MrBayes is shown in Fig. 3a. In this tree, the *O. felineus* sequences diverge earlier than those of *O. viverrini* and *C. sinensis.*

The length of the branch running from the Opisthorchiidae common ancestor to the node corresponding to the common ancestor of *C. sinensis* and *O. viverrini* is shorter in both trees as compared with the branches leading to their tops. PhyloBayes estimates the support value for the *C. sinensis*–*O. viverrini* clade as 1 versus 0.89 given by MrBayes and 98, the bootstrap value given by RAxML. Thus, *C. sinensis* and *O. viverrini* diverged almost immediately after *O. felineus* was separated from the common ancestor of these three liver fluke species.

### Analysis of pre-mRNA processing revealed many trans-spliced genes

We used a combination of several gene finding approaches to refine a reliable annotation of protein-coding genes in *O. felineus* genome (Additional file 1: Figure S2; Additional file 2: Table S5). The RNA-seq data for two life stages (metacercaria and adult) allowed for a total of 11,455 protein-coding genes and 21,036 their mRNA products to be identified (Additional file 2: Table S2). The number of found genes is less than that predicted in *C. sinensis* and *O. viverrini* genomes [15, 16]. Nevertheless, this difference is not much informative, since it was attributed mainly to the strictness of filtering invalid or insufficiently supported gene models, the initial number of which was quite large (Additional file 2: Table S5). In fact, the comparative evaluation of the three genome annotations using BUSCO software (Additional file 2: Table S10) as well as the results of the orthology inference (Fig. 7a, discussed further) showed that the applied filters did not hamper the completeness of *O. felineus* annotation.

**Fig. 4:**
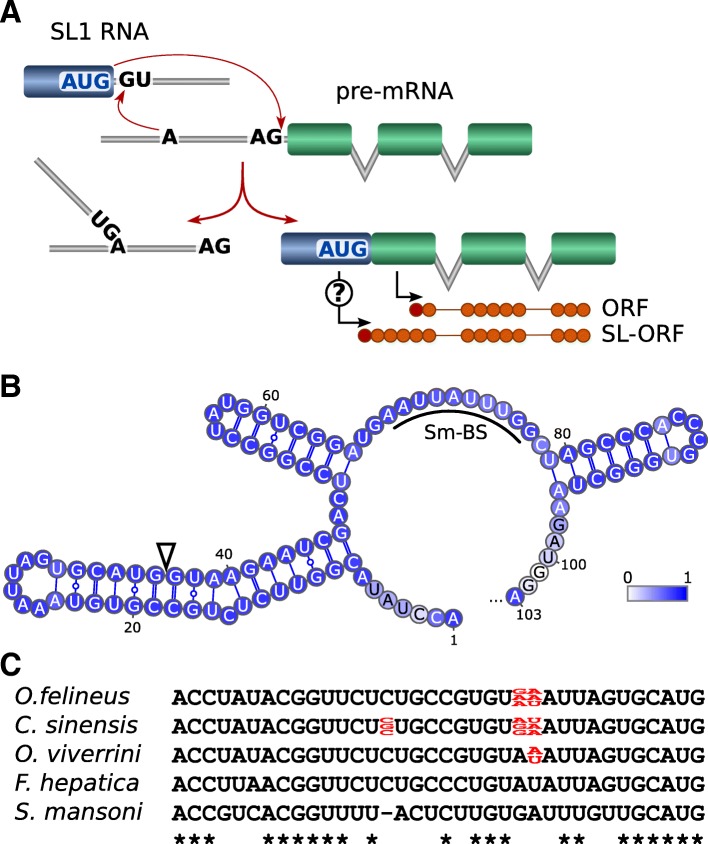
Identification of SL1 RNA in *O. felineus*. **a**. Schematic representation of SL-dependent trans-splicing in flatworms. A potential role of SL-derived AUG codon in translation of the trans-spliced transcript is denoted by a question mark. **b**. The predicted secondary structure of *O. felineus* SL1 RNA composed of three stem-loops and containing Sm protein-binding site (Sm-BS). The color scale depicts base-pair probabilities. Splice donor site is marked by a triangle. Prediction was performed using RNAfold WebServer (http://rna.tbi.univie.ac.at/). **c**. An alignment of SL1 exons identified in three Opisthorchiidae species, *F. hepatica* and *S. mansoni*. Intraspecific sequence variations are marked in red

When analyzing the RNA-seq data, we found that trans-splicing is broadly involved in expression control of *O. felineus* genes. This feature of pre-mRNA processing was not described earlier for Opisthorchiidae liver flukes. Trans-splicing (TS) is a special form of pre-mRNA processing when exons from two different primary RNA transcripts are joined. One of its common types is spliced leader trans-splicing, which results in addition of a capped noncoding spliced leader (SL) sequence to the mRNA 5′ ends by a mechanism very similar to cis-splicing (Fig. 4a). This mechanism of RNA editing occurs in ~ 70% of the genes of the round worm *C. elegans* [22] and almost all genes of *Trypanosoma brucei* [23] versus the flatworm *S. mansoni,* shown to have only 11% of the genes the transcription of which is coupled with trans-splicing [24].

We have determined the potential SL sequences from raw transcriptome data for the three opisthorchiids as well as for *F. hepatica* and *S. mansoni* genomes. The predicted secondary structure of *O. felineus* SL1 RNA composed of three stem-loops and containing Sm protein-binding site is shown (Fig. 4b). The SL1 RNA candidates were further approved by the similarity of primary and secondary structures to the known SL sequences (Fig. 4c). Interestingly, several polymorphic positions are present in 36-nt SL sequences of all three opisthorchiids but not in *F. hepatica* and *S. mansoni* (Fig. 4c).

As is evident from the genomic data, the *O. felineus* SL RNA is encoded by more than 300 copies of a 920-bp long sequence arranged in tandem repeats. We have annotated the trans-splicing sites for the whole set of annotated protein-coding genes using all available RNA-seq data. As was shown, the products of 6905 (61%) genes contain SL sequence; however, more stringent filtering by mapping quality and fragment coverage reduced this number to 5414 (47%) genes bearing 10,805 spliced leader trans-splicing (SLTS) sites, which were used in further analysis. However, the list of affected genes may yet be broader, since our RNA-seq-based approach tend to underpredict SLTS because of the under-representation of **5′**-ends of transcripts in poly(A)-enriched RNA-seq libraries as well as because of the skipping too short SL sequences in homology search. Thus, we have for the first time demonstrated that the products of almost half *O. felineus* genes contain an SL sequence. This suggests an important role of trans-splicing in the processing of RNA in liver flukes.

### High degree of evolutionary conservation of SLTS in flatworms

**Fig. 5:**
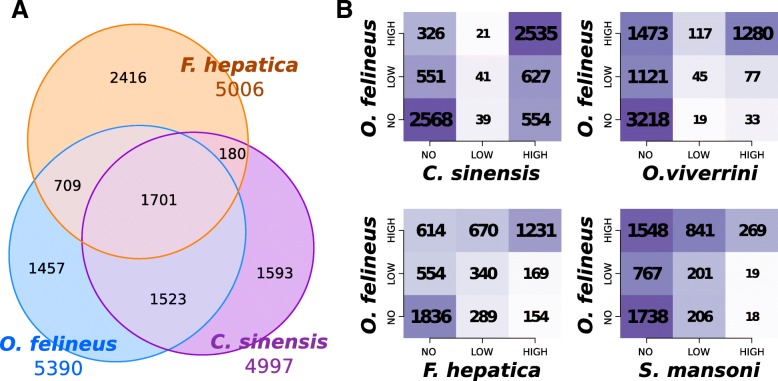
Genome-wide distribution and interspecies conservation of splice-leader trans-splicing (SLTS) in *O. felineus*. **a**. A Venn-diagram showing the overlap between the clusters of orthologous genes bearing SLTS sites in *O. felineus*, *C. sinensis*, and *F. hepatica*. **b**. Interspecies differences of SLTS, illustrated by pairwise comparison tables of orthologous genes grouped by SLTS efficiency (NO, not detected; LOW, < 50%; and HIGH, > = 50%); both font size and colour intensity correspond to the provided number of orthologous clusters

To further characterize the conservation of transcriptome trans-splicing events within the Trematoda and analyze its possible evolutionary lability, we have also analyzed the distribution of SL-TSSs sites within the targeted pre-mRNAs of *O. viverrini* and *C. sinensis*, as well as in more distant relatives, *F. hepatica* and *S. mansoni*. The results of comparison of orthologous genes sharing SLTS are shown as Venn diagram and tables (Fig. 5a, b). A high level of conservation of highly efficient SLTS sites is observed in *O. felineus*, *C. sinensis,* and *F. hepatica* genes, with most differences attributed to inaccuracy or inconsistency of gene annotations (Fig. 5b). The SLTS events in *O. viverrini* were highly underestimated owing to an insufficient depth of available RNA-seq data. However, only 15% of the genes were found to be trans-spliced in *S. mansoni*, which is consistent with the previous studies [24]. Thus, the trans-splicing machinery of schistosomes has considerably diverged from the remaining studied species, as is evident from the primary SL-RNA structure as well as the conservation and overall occurrence rate of SLTS events.

### Trematode genomes include many microintrons

**Fig. 6:**
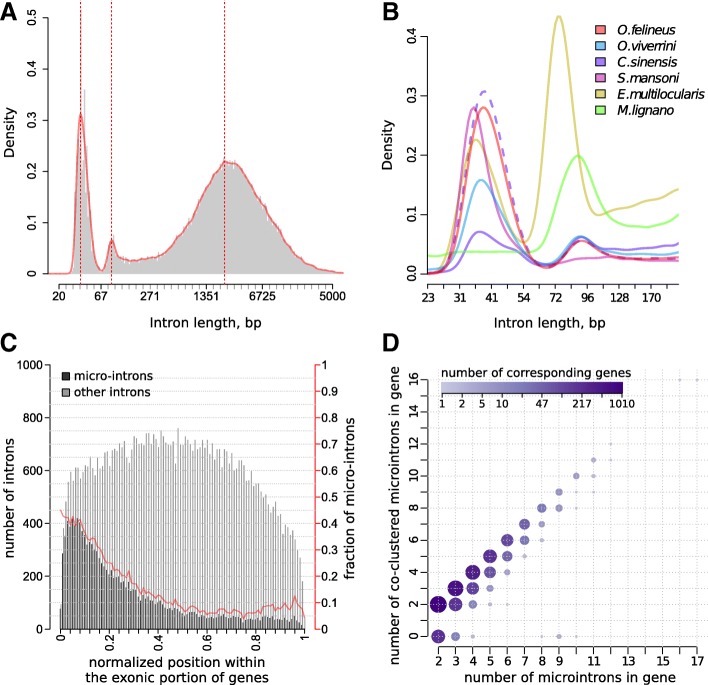
Microintrons are preferentially located near the transcription start site and cluster together. **a**. Intron length distribution in *O. felineus* genome. **b**. A bimodal distribution of microintrons in flatworm genomes. The dashed line represents intron distribution in *C. sinensis* transcriptome assembly of raw RNA-seq data. **c**. Relative localization of introns within the *O. felineus* genes. Red line and scale represent a fraction of micro-introns among all introns. **d**. Microintrons tend to cluster together within *O. felineus* genes

Analysis of the lengths of introns in the *O. felineus* genome demonstrates that the length distribution has not only a large characteristic peak at 3000 bp, but also two additional peaks with maximums at 37 and 90 bp (Fig. 6a). Ultra-short introns, or microintrons, with a length of < 75 bp account for approximately 34% of all annotated introns and are contained in 4997 (44%) genes.

The nucleotide sequences of *O. viverrini, C. sinensis, S. mansoni* (blood fluke), *Echinococcus multilocularis* (tapeworm), and *Macrostomum lignano* (free-living flatworm) genomes were also analyzed in a similar way. The fraction of microintrons with a varying average length is also observable in other flatworms, including the free-living species (Fig. 6b). Microintrons can be underrepresented in draft gene annotations because of usually too high default intron length threshold in the annotation software as in the case of *C. sinensis* annotation (Fig. 6b).

Analysis of the overrepresented motifs in microintron sequences has not found any motifs unique for these introns. We have shown that microintrons have more precise (closer to the consensus) splicing sites as compared with the other introns but frequently lose other splicing signals as compared with the remaining introns, including the polypyrimidine tract. The distribution of microintrons in the *O. felineus* genes also has certain specific features. First, when a gene contains several microintrons, they, as a rule, are adjacent to each other, forming clusters (Fig. 6d). Second, microintrons more frequently occupy the 5′-end of genes (Fig. 6c). These features suggest that this class of introns has separate functional significance in transcription and processing mechanisms.

### Pathogenesis related genes are differentially expressed between trematode species

We have earlier published the results of studying the transcriptome on the Illumina platform for metacercaria and adult stages of *O. felineus* [18]. Here we expanded this study by adding two libraries for metacercaria and one library for adult stage, thereby achieving three biological replicates per condition (Additional file 2: Table S6). Since we have done more advanced gene annotation as compared with the earlier results of de novo transcriptome assembly and increased the number of biological replicates for both life stages, we have revisited the differential expression of genes for these stages. Most results were consistent with the earlier data [18]. At the metacercaria stage, highly transcribed genes include ribosomal proteins (18 out of 37 of the highly transcribed genes) (Additional file 2: Table S6). Overall, the metacercaria mainly transcribes housekeeping genes, for example, ribosomal proteins, heat shock proteins and ubiquitin. In the adult stage, highly transcribed genes included tubulins, egg protein and glutathione transferases.

**Fig. 7:**
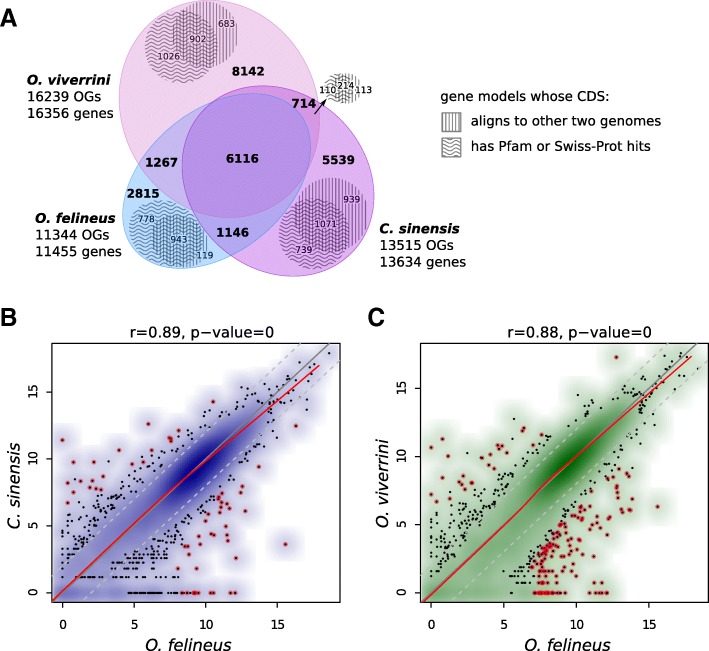
Interspecies comparison of gene repertoire and expression among adult *O. felineus*, *O. viverrini*, and *C. sinensis*. **a**. Proportional Venn-diagram of orthologous clusters identified in three species by ProteinOrtho. Non-orthologous genes that match to Swiss-Prot/Pfam datasets or genomes of other two flukes are shown as hatched areas. **b**. Differences in gene expression of adult worms. The genes with expression values differing more than fourfold (*p* < 0.01) are colored dark red (OF, *O. felineus;* OV, *O. viverrini;* CS, *C. sinensis*). The Pearson correlation coefficient for the pair *O. viverrini*–*O. felineus* was *r* = 0.89 (*p*-value = 0); for the pair *O. felineus*–*C. sinensis* was *r* = 0.88 (*p*-value = 0)

Of further interest was the data on interspecies comparison of transcriptomes, namely, of adult *O. felineus, O. viverrini*, and *C. sinensis*. Since this task requires a reliable inference of orthologous relations between the annotated genes, we applied ProteinOrtho synteny-aware algorithm on protein sequences, extracted from the available gene annotations for five species under study (Fig. 7a, Additional file 2: Table S7). The orthology table for the three opisthorchiids was extracted and analyzed in detail (Fig. 7a). As a result, only 6116 clusters of orthologous genes were found in all three species, while substantial portion of genes of each species had no orthologs at all. Since the latter might be enriched with false-discoveries (e.g., transposons, non-coding transcripts, etc.), we searched them for homology to annotated Pfam domains or Swiss-prot proteins. For each species, about two thousand non-orthologous genes had matches to Pfam/Swiss-prot databases; although the fraction of the unmached genes was considerably smaller for *O. felineus* annotation compared to the other ones. Taken together, the orthology analysis showed that all three annotations suffer from completeness much more than their corresponding draft genomes. Moreover, a notable fraction of orthologous genes in each species is incomplete because of the fragmented assemblies or annotation errors, making the inferred orthologous groups inconsistent in terms of quantitative comparisons.

To overcome some of these problems, we decided to (i) exclude all annotations except one from orthology analysis and use instead the whole genomes to search for reciprocal-best homologs of *O. felineus* annotated genes, and (ii) refine the exact regions inside the genes that are shared among the species, thereby counteracting the incompleteness of both repertoire and content of gene models. The workflow (as described in section *Interspecies comparison of gene expression*) allowed us to identify the nearly-identical ‘orthologous’ coding sequences for 9952 (87%) and 10,077 (88%) genes for the comparisons to *O. viverrini* and *C. sinensis*, respectively. As has emerged, expression of most genes of these three opisthorchiid species was highly consistent in spite of the completely different sources of RNA-seq data (Fig. 7b,c; Additional file 2: Tables S8, S9). Since the three considered opisthorchiid species have sufficiently distinct environmental differences [9], these results may seem unexpected.

Nonetheless, a number of genes of adult *O. felineus, O. viverrini*, and *C. sinensis* have a significantly different level of expression (Fig. 7). In total, 61 such genes were recorded for the pair *O. viverrini*–*O. felineus* (Fig. 7a; Additional file 2: Table S8) and 160, for *O. felineus*–*C. sinensis* (Fig. 7b, Additional file 2: Table S9). Using InterPro protein sequence analysis [pfam (http://pfam.xfam.org/) [25], CDD [26], and Prosite [27]], we tried to find particular groups of genes enriched within the differentially expressed genes.

Kunitz-type inhibitors (3 out of 14 genes in the genome) were observed among the genes differentially expressed in *O. felineus* and *C. sinensis*. The expression of these three genes in *O. felineus* exceeded the expression of the homologs in *C. sinensis* more than 20–100-fold. Another group of differentially expressed genes were CAP domain genes (4 genes of 27, namely, cysteine-rich secretory proteins, antigen 5, and pathogenesis-related 1 proteins). Their expression in *O. felineus* was 50–200-fold higher as compared with the homologous *C. sinensis* genes. The group of differentially expressed genes for the pair *O. felineus*–*O. viverrini* also includes the genes encoding ribosomal proteins L19 (two genes out of two in the genome), papain family cysteine proteases (2 genes out of 25) and glyceraldehyde 3-phosphate dehydrogenase (one out of three).

Interestingly, products of majority of the differentially expressed genes found in our study contain domains characteristic for helminth-secreted proteins. In particular, CAP protein family (cysteine-rich secretory proteins, antigen 5, and pathogenesis-related 1 proteins), papain family cysteine proteases and Kunitz-type inhibitors are among the most represented compounds of secretome across 44 helminth species [28]. The properties of several gene families selected by their relevance in the pathogenesis of opisthorchiasis are discussed below.

### Detoxification network genes were found in the genome

The detoxification system of the liver fluke is of special interest, since its components are promising pharmacological targets [29–32]. Detoxification system is essential for both the adaptation to host environment and survival of the parasite [30, 31]. In addition, parasite’s detoxification system is most likely responsible for synthesis of parasite-specific genotoxic metabolites of cholesterol, recently discovered in *O. felineus* and other carcinogenic trematodes, *S. haematobium* and *O. viverrini* [33, 34].

The proteins putatively involved in oxidation and reduction of substrates form the group of enzymes prevalently implementing phase I metabolism of exogenous substrates and are the most important component of detoxification system in all organisms. Cytochromes P450 (CYPs) are among these proteins. CYPs family in parasitic and free-living flatworms is drastically different: the free-living species have dozens of diverged CYP genes (39 CYPs in *Schmidtea mediterranea*), whereas the parasitic species (Schistosomatidae, Opisthorchiidae, Taeniidae, and Fasciolidae) most likely have only one cytochrome P450 [30, 35]. *O. felineus* CYP is involved in the metabolism of exogenous substrates, is important for survival of adult individuals, and represents a promising target for anthelminthic therapy [30, 32]. Differential expression of this gene was consistent with the earlier data [30]. Furthemore, *O. felineus* detoxification phase I is also represented by the genes encoding aldo-keto reductases, aldehyde dehydrogenases, and alcohol dehydrogenases (Additional file 2: Table S6). Expression of all aldehyde dehydrogenases was higher in adult stage, than in metacercariae; while the expression of aldo-keto reductases was almost on the same level in both stages. Interestingly, the liver flukes lack the group of enzymes with a monooxygenase activity (analogous to CYPs), namely, flavin monooxygenases (Pfam00743). Moreover, we failed to find any flavin monooxygenase sequences in any parasitic flatworm genomes.

Phase II enzymes are represented, in particular, by glutathione peroxidase (GPx) and glutathione-S-transferase (GST). The *O. felineus* genome contains six GST genes, which display the highest expression among all detoxification genes. The 28 kDa GST sigma gene (CRM22_011285 in Additional file 1: Figure S5) is especially active; its expression in the adult worm is by two–three orders of magnitude higher as compared with the other detoxification genes.

UGTs, common phase II xenobiotic metabolism enzymes in vertebrates, enhance hydrophilicity and availability of substrates to efflux transporters. The UGT superfamily comprises two families (UGT1 and UGT2) and over 20 isozymes. UGTs play a significant role in drug resistance of helminths [29]. Currently, 34 UGT genes are known in the nematode *Haemonchus contortus* genome [36] and 72 UGT genes, in *C. elegans* genome [37]. We failed to find any UGT genes in the *O. felineus* genome as well as in the genomes of other Opisthorchiidae and Schistosomatidae. In addition, we did not find any arylamine N-acetyltransferases (PF00797), which are also common phase II enzymes in vertebrates. Thus, the detoxification phase II of parasitic flatworms has some features distinguishing it from the corresponding systems of the other organisms, including the host.

The detoxification phase III is represented by membrane efflux transporters, protecting organisms from external toxic compounds, including drugs. Five distinct families of the efflux transporters are recognized. The most frequently studied are ABC transporters, for example, ABCB1 (also known as P-glycoprotein or multidrug resistance protein 1, MDR1), ABCC1 (multidrug resistance associated protein, MRP1), and ABCG2 (also known as breast cancer resistance) [38, 39]. The *O. felineus* genome contains 23 genes encoding ABC transporters. Four of ABC genes are homologous to human P-glycoprotein (Additional file 1: Figure S5). Differential expression of these genes was consistent with the earlier data [40].

### Dozens of NPC2 genes were revealed in Opisthorchiidae genomes

One of the specific features of genomes of closely related species is the number of copies of orthologous genes. The NPC2 genes (Niemann–Pick disease type C2) encode the proteins that are potentially involved in intracellular and extracellular transport of sterols. We have discovered 48 NPC2 genes in the *O. felineus* genome. Most eukaryotes have only one NPC2 gene. Earlier, 25 genes coding for NPC2-like proteins have been detected in the *O. viverrini* and *C. sinensis* genomes [16]. The function of these proteins in helminths is vague. There is evidence suggesting the chemosensory role for these proteins in Arthropodae [41]. Such diversity of these proteins in the liver flukes might be determined by the life activities of these helminths, i.e., their function may putatively consist in binding and transporting the host lipids and sterols for the adult helminth metabolism. However, comparative transcriptomic analysis of the *O. felineus* metacercariae and adult has shown that 43 of the observed 48 NPC2 proteins are differentially expressed in metacercariae rather than in adult worm (Additional file 2: Table S6). Expression of only two NPC2-like genes is specific of the adult worm stage. Thus, the NPC2-like proteins in general are not crucially important for the life activities of an adult individual and potentially are not associated with the lipid binding and transport in an adult individual living in the bile. The biological significance of increasing the number of NPC2-like genes in the *O. felineus* genome compared to genomes of *O. viverrini* and *C. sinensis* remains to be revealed.

### Granulin-like growth factors are involved in parasite–host interactions

Granulins, the growth factors secreted by liver flukes, are involved in the development of cholangiocarcinoma [42]. As has been shown, the *O. viverrini* granulin (*Ov-*GRN-1) stimulates proliferation of cholangiocytes [42]. The properties of *O. felineus* granulins are yet vague.

**Fig. 8:**
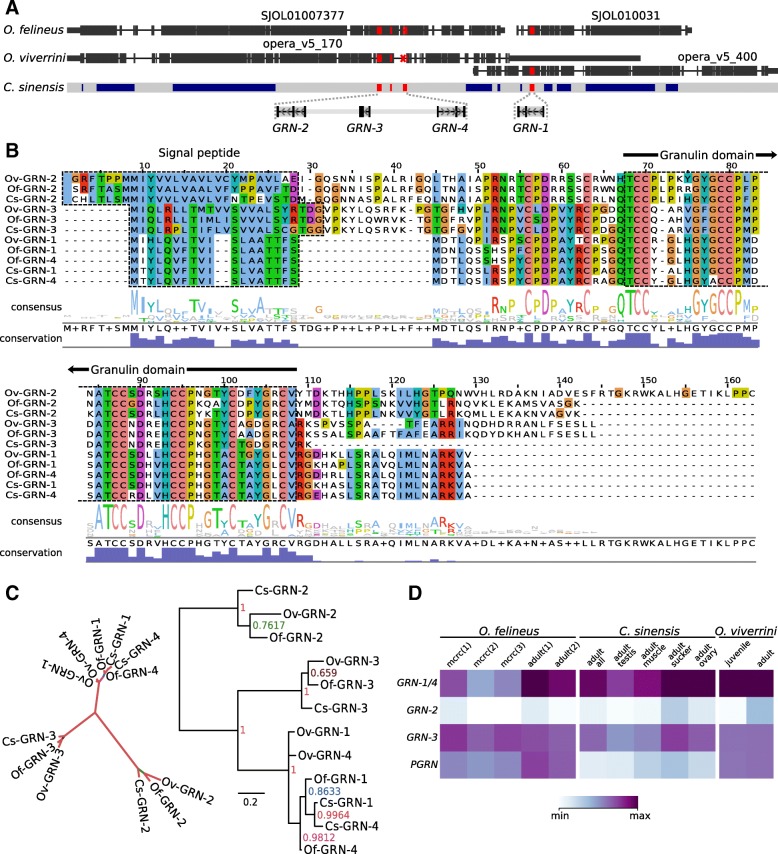
Granulin-like genes in Opisthorchiidae genomes. **a**. Granulin-like genes are co-localized to one locus in three opisthorchiid genomes. Alignment (**b**) and cladogram (**c**) of granulin-like proteins are presented. **d**. Heatmap of granulin-like gene expression in three Opisthorchiidae species

Four genes (GRN-1–GRN-4) coding for single domain granulins have been detected in the three studied opisthorchiid genomes as well as one gene of a multidomain progranulin, PGRN (Fig. 8). The genes of single domain granulins are localized to a single chromosome locus and form a syntenic gene group in the three studied genomes (Fig. 8a). The GRN-4 gene displays 95% identity to the nucleotide sequence of GRN-1 gene (Fig. 8b), the product of which has a confirmed mitogenic activity [42]. Presumably, this duplication is fixed in the opisthorchiid genomes owing to a considerable functional importance of the corresponding product in the host–parasite interaction. *Ov*-GRN-4 gene in the *O. viverrini* genome is fragmented (Fig. 8a), possibly, because of genome misassembly. The earlier identified *O. viverrini* gene encoding GRN-2 and GRN-3 (which we have earlier detected in the *O. felineus* transcriptome; GBJA01006896.1) display high interspecific conservation but still are considerably different from both GRN-1/GRN-4 and one another (Fig. 8c). The observed stage specificity in expression of granulins demonstrates that the GRN-1/GRN-4 proteins are mainly expressed in adult individuals (Fig. 8d), while GRN-2 and/or GRN-3, almost not expressed in adult parasites, might be functionally involved in the parasite–host interaction in two other hosts (mollusks and fish). In particular, it is now known that granulin-like proteins are involved in the parasite–host interaction of trematodes and mollusks: the granulin of the snail *Biomphalaria glabrata* (BgGRN) activates proliferation of hemocytes, thereby providing resistance to *S. mansoni* infection [43].

## Discussion

The draft *O. felineus* genome size is approximately 684 Mbp, being slightly longer as compared with *С. sinensis* and almost the same as the *O. viverrini* genome; and all three genomes have very similar content and diversity of repetitive elements. The total number of the predicted *O. felineus* protein-coding genes is approximately by one-third smaller as compared with *O. viverrini* and *C. sinensis* [16, 17]. However, this difference is determined rather by stringent filtering criteria for selecting final protein-coding models used in this work than the completeness of *O. felineus* genome or bona fide loss of genes. Actually, the obtained *O. felineus gene* annotation showed even superior indicators of completeness in BUSCO evaluation (Additional file 2: Table S10) and presumably lower false-discovery rate, as suggested from orthology analysis (Fig. 7a). In addition, we used the RNA-seq data obtained by sequencing the transcriptomes of two liver fluke forms—adult individual and metacercaria—which in our views increases the reliability of the data. Anyway, the orthology analysis demonstrated that the completeness of both draft genomes and their gene annotations are far from being comprehensive for all three species.

We observed both noticeably high heterozygosity of the sequenced individual and substantial genetic diversity in pooled samples. Since we sequenced the environmental sample taken from natural population, it was quite possible that we would face with high levels of sequence divergence. In our case, the latter is unlikely to have originated from variation of ploidy levels or host contamination. The cytogenetic studies of *O. felineus* have shown that its karyotype is stable and consists of 2n = 14 chromosomes [14, 44] Similarly, our data on genome assembly and gene content also revealed no perceptible evidence of anomalous ploidy levels or excess of contaminating sequences.

High genetic diversity of *O. felineus* is consistent with previous studies. In particular, the *O. felineus* specimens endemic to Western Siberia differ in their susceptibility to praziquantel [45], an anthelmintic drug used to treat opisthorchiasis, clonorchiasis, schistosomiasis, and other diseases caused by trematodes [4, 46]. Our results comply with the data on high heterozygosity in the organisms that pass through a certain stage of asexual reproduction in their life cycle [47]. In addition, relatively high polymorphic rates between and within individuals were already observed in some other Trematoda species, such as *F. hepatica* [19] and *S. mansoni* [24]. Interestingly, *O. felineus* considerably differs from *O. viverrini* in the level of heterozygosity. Indeed, the sequencing data of a pooled sample comprising 25 adult *O. viverrini* individuals lacked similarly high heterogeneity [16]. We assume that these differences can stem from effective population size and the specific features of *O. felineus* and *O. viverrini* foci, and may indicate a higher potency of *O. felineus* population for rapid adaptive response to control and preventive measures of opisthorchiasis.

Analysis of the genome-wide synteny between *O. felineus, O. viverrini,* and *C. sinensis* has allowed us to demonstrate a considerable variation in the liver fluke genomes. Similar results were obtained by Young et al. [16]. However, our results suggest that a structural similarity between the *O. felineus* and *C. sinensis* genomes is higher as compared with that of *O. viverrini* to *O. felineus* and to *C. sinensis.* These data match well the results of karyotyping: *O. felineus* and *C. sinensis* have seven pairs of chromosomes versus *O. viverrini,* carrying six chromosome pairs [14, 44].

The phylogenetic relationships between the genera Opisthorchis and Clonorchis is controversal [48]. Kang et al. [49] used the ITS1 sequences from *O. felineus*, *O. viverrini* and *C. sinensis*, and demonstrated that *C. sinensis* is sister group to *O. viverrini* and *O. felineus*. Analysis of the phylogenetic relationship between ITS2 and mitochondrial *cox1* showed that *O. felineus* was more closely related to *C. sinensis* than to *O. viverrini* [50]. Similar relationship was demonstrated using *cox1* sequences by Saijuntha et al. [51], *cox1*, *nad1* and paramyosin gene (Pm-int9) sequences by Pitaksakulrat et al. [52]. The same topology of phylogenetic tree between *O. felineus*, *C. sinensis* and *O. viverrini* obtained by analysis of concatenated amino acid sequences from mitochondrial protein-coding genes by Wang et al. [53]. and Liu et al. [54].

In our work analysis of 1563 protein families suggested the phylogenetic tree topology with *C. sinensis* and *O. viverrini* species grouping together and *O. felineus* representing a sister group. Our results are in agreement with the phylogenetic tree estimated using paramyosin gene (Pm-int9) [55], mitochondrial amino acid sequences analyzed by Cai et al. [56], ribosomal proteins [18].

Taken together, the results of analysis of the synteny between three opisthorchiid species and of their phylogenetic relationships demonstrate that *O. felineus* and *C. sinensis* are closely related and do not support separation of *C. sinensis* from the genus *Opisthorchis.* Presumably, *C. sinensis* occupies an intermediate position between *O. felineus* and *O. viverrini*. The geographic vector of distribution of opisthorchiasis and clonorchiasis foci also favors this hypothesis. The opisthorchiasis caused by *O. felineus* has been recorded in many European countries; however, the most intense foci of this disease are in North Asia, namely, in Russia and Kazakhstan [3]. Clonorchiasis is mainly spread in the Russian Far East, China, Korea, Japan, and to a lesser degree in Laos and Vietnam [10]. The main foci of *O. viverrini* opisthorchiasis are in the Southeast Asia, namely, in Thailand, Laos, Vietnam, and Cambodia [8].

We have found that all three studied opisthorchiidae species are characterized by much more extensive involvement of trans-splicing in RNA processing compared to the most well-studied trematode, *Schistosoma mansoni* [24]. Since trans-splicing was found to occur with high efficiency in these species, the affected 5` ends of many transcripts tend to generate spurious alignments to the reference genome, leading to incorrect prediction of gene structure and even the corresponding structure of encoded protein as a result of the standard genome annotation workflow. Thereafter, accounting properly the trans-splicing events in transcriptomes along with another important feature of gene organization in flatworms, microintrons, resulted in a much more reliable annotation of genes and their protein products in *O. felineus,* e.g. the resolution of polycistronic gene products.

The bimodality of intron length distributions has been observed also in many eukaryotic species. Two classes of introns, with a peak of short introns or microintrons and a much flatter peak of longer introns ranging up to thousands of base pairs, are present in humans, *Arabidopsis thaliana*, *Drosophila melanogaster*, and *C. elegans* [57]. Some Ciliates (Paramecium) contain microintrons with a length divisible particular, 33–35, 47–51, and 78–80 bp [58]. Two peaks of short introns has been earlier described in parasitc flatworms, including Cestoda [59] and Monogenea species [60]. However, we have found that this feature is of the same relevance for opisthorchiidae species. The peculiarities of microintrons revealed in the current study suggest that the apartness of this fraction of introns is maintained by their functional significance in transcription and processing mechanisms. For example, their clustering at the 5′ ends of pre-mRNAs may be driven by a preference of an intron definition mechanism over exon definition.

There are some difficulties in comparative analysis of gene content and expression between three liver flukes, since the existing genome assemblies are draft, and the corresponding gene annotations are far from being complete and compatible. However, we have made an effort to generate a congruent set of orthologous expression units based on *O. felineus* annotation only. While expression levels of a number of genes showed remarkable differences between the species, the overall expression profiles were highly consistent across the compared species, suggesting a high similarity of all biological pathways in adult liver flukes that colonize the bile ducts of mammals.

## Conclusions

Lack of *O. felineus* genomic data is an obstacle to the development of comparative molecular biological approaches necessary to obtain new knowledge about the biology of epidemiologically important Opisthorchiidae trematodes, to identify essential pathways linked to parasite-host interaction, to predict genes that contribute to liver fluke pathogenesis and for the effective prevention and control of the disease. Here we present the first draft genome assembly of *O. felineus* and its gene repertoire accompanied by a comparative analysis with that of *O. viverrini* and *Clonorchis sinensis*.

This study contribute to comparative genomics of flatworms, molecular mechanisms of RNA processing in helminths and evolutionary history of Opisthorchiidae trematodes. The availability of *O. felineus* genome we provide, and comparative transcriptomics data will help support the development of novel drugs and vaccines for the treatment and prevention of liver fluke infection.

## Methods

### Ethical statement

All of the procedures were in compliance with The Code of Ethics of the World Medical Association (Declaration of Helsinki) for animal experiments http://ec.europa.eu/environment/chemicals/lab_animals/legislation_en.htm. The animals were kept and treated according to the protocols approved by the Committee on the Ethics of Animal Experiments with the Institute of Cytology and Genetics (Permit Number 7 of November 19, 2011).

### Study design

Two thousands of *O. felineus* metacercariae were collected from naturally infected fish (*Leuciscus idus*) caught in the Ob River near Novosibirsk (Western Siberia) and routinely extracted [45, 61]. Territories where sample collection (fishing) took place were neither conservation areas nor private, nor otherwise protected; hence, no fishing permits were required. The fish species collected are not considered endangered or rare, and the fishing methods complied with the Federal Law N166-F3 of 20.12.2004 (ed. 18.07.2011), “Fishing and conservation of water bio-resources”. Syrian hamsters (*Mesocricetus auratus*) were purchased from the Animal Breeding Facility with the Institute of Cytology and Genetics, Siberian Branch, Russian Academy of Sciences. Five hamsters aged 6 to 8 weeks were orally infected with 75 *O. felineus* metacercariae. Euthanasia was performed using carbon dioxide, and all efforts were made to minimize suffering. The animals were maintained in their home cage while CO_2_ was induced at a flow 10% per minute. Adult flukes were recovered from the hepatobiliary tract of hamsters three months after the infection, pooled and thoroughly washed with saline.

### List of data used for the interspecies analysis

To analyze transcriptomic data Sequence Read Archives (SRA) were taken from NCBI https://www.ncbi.nlm.nih.gov/sra in the research: ERR604978-ERR604981, SRR189060 for *C. sinensis*; SRR497632, SRR497633 for *O viverrini*; ERR576952, ERR576954, ERR576956, ERR576958, ERR576968 for *F hepatica*; ERR1328228-ERR1328233, ERR1328243, ERR1328260-ERR1328267 for *S mansoni.* Genome assemblies and gene annotations were taken from WormBase ParaSite (version WBPS6) [62, 63]: PRJNA222628 (*O. viverrini*) [16], PRJDA72781 (*C. sinensis*) [17], PRJEA36577 (*S. mansoni*) [24, 64], PRJEB122 (*E. multilocularis*) [59], PRJNA284736 (*M. lignano*) [65], *F. hepatica* (PRJEB6687) [19].

### Genome sequencing and data processing

Genomic DNA (gDNA) was isolated from using proteinase K digestion following phenol–chloroform extraction. For short insert libraries, the gDNA isolated from a randomly chosen single adult *O. felineus* worm was fragmented in a Covaris S2 (Covaris™, United States) sonicator to an average fragment size of 200–500 bp and the sizes selected on Pepin Prep (Sage Science, Inc., United States) for the insert were 180 and 270 bp. Construction of genome libraries and all subsequent manipulations were performed using paired-end library preparation kit in accordance with the manufacturer’s protocols (Illumina, United States). Genome libraries were suitable for paired-end reading. Sequencing was performed on Illumina Genome Analyzer II (GAII) and HiSeq1500 (Illumina, United States) platforms. For mate-pair libraries, the gDNA was isolated from the pool of 50 randomly chosen worms, and Nextera Mate Pair Sample Preparation Kit (Illumina, United States) was used according to manufacturer’s protocols. Three different insert size libraries of 3, 5, 8, and 10 kb were sequenced on Illumina HiSeq 1500 (Illumina, United States).

TRIzol® reagent was used for total RNA extraction from the pooled adult (randomly chosen 50 adult worms) and the pooled metacercaria (1000 worms) homogenized tissue using standard protocol (Invitrogen, United States). The RNA concentration in samples was measured using BioAnalyzer 2100 (RNA 6000 Nano Kit; Agilent, US). The mRNA-seq libraries (cDNA libraries) were prepared with mRNA-Seq Sample Preparation kit (Illumina) according to the manufacturer’s instructions. The cDNA libraries were sequenced in an Illumina Genome Analyzer II (GAII) DNA Sequencing Platform (Illumina, United States).

### Genome assembly

Raw sequencing data were preprocessed (Additional file 1: Figure S1) by removing low-quality base-calls and adapter sequences and potential PCR duplicates using cutadapt v1.18 [66]. Mate-pair libraries were also filtered on a substantial read pair overlap determined by pear read-merging statistical algorithm [67]. The resulting reads were initially assembled using Allpaths-LG [68] software in a with ‘haploidify’ mode to overcome a high level of genome heterozygosity evident from k-mer frequency distribution (Fig. 2). Additional scaffolding with jumping libraries was performed using the BESST [69] program and the remaining gaps were partially filled by applying SOAPdenovo GapCloser tool [70]. A small fraction of short redundant contigs showing > 95% homology and reduced read coverage was filtered out from the assembly. Finally, three iterations of iCORN2 algorithm from PAGIT toolkit [71] were performed to correct 1–3 bp errors in the assembly. Final assembly was evaluated using REAPR ([72], Additional file 2: Table S11) and BUSCO ([73], Additional file 2: Table S10) tools.

### Variant calling

Variant calling was performed using the GATK pipeline. Two libraries (both prepared from the same single individual worm) were used as technical replicates. Initial calling was done by HaplotypeCaller module in -ERC(GVCF) mode, and the results were subjected to GenotypeGVCFs module for joint genotyping. Indels were hard-filtered according to the GATK best practices recommendations. Hard filtering of the resulting SNPs was performed using the stricter criteria QD < 5.0, FS > 40.0, MQ < 35.0, MQRankSum < − 2.5, SOR > 2.5, ReadPosRankSum < − 4.0, and DP < 150 or DP < 90 (depending on the library analyzed). An intersection of the two technically repeated variant annotations was finally taken (resulting in 3,723,880 SNPs and 302,377 indels). Further analysis of the heterozygosity rate was limited to genomic intervals with fragment coverage lying between 0.75 and 1.25 of the median coverage in order to reduce the impact of potential assembly and mapping errors (collapsed or duplicated sequences). Heterozygous variants falling into these intervals were counted and divided by the total length of the intervals.

### Genome synteny

The examined genomes were compared using the scheme implemented by Young et al. [16]. Genomic scaffolds were aligned using the nucmer (nucleotide similarity) or promer (amino acid similarity) tools with the program MUMmer v. 3.23 [74] to assess genome-wide similarities.

The genomic sequences of *O. felineus, O. viverrini, C. sinensis,* and *S. mansoni* were compared in a pairwise manner. The sequences were aligned with the help of MUMmer v. 3.23 [74]. First, the *O. felineus, O. viverrini,* and *C. sinensis* scaffolds with a length over 1000 bp were aligned at the level of amino acid sequences by the promer program (parameters: –maxgap = 500 –c 20; unique pairwise alignments were filtered with the delta-filter program with parameters –r –q) to *S. mansoni* chromosome 1 (Smp.Chr_1), used as a reference. The syntenic blocks were identified in the constructed alignments with SyMap v. 4.2 [21]. Second, the *O. viverrini* and *C. sinensis* scaffolds were aligned at the amino acid sequence level (the promer program with the above listed parameters) to SJOL01009849 sequence of *O. felineus*, which was the longest one (3238 362 bp). Third, the *O. viverrini* and *C. sinensis* scaffold sequences with a length over 1000 bp were aligned to the *O. felineus* scaffolds using the nucmer program (parameters: –maxgap = 500 –c 100, delta filter –r –q). Then 10 *O. felineus* scaffolds that had the largest coverage by *O. viverrini* and *C. sinensis* sequences according to the nucleotide alignment were selected. These sequences were analyzed using the SyMap program together with the homologous opisthorchiid scaffolds. The analysis was conducted for both the sequences with unmasked repeats and the genome sequences with repeats masked with N symbols.

Additionally we used OrthoCluster software to calculate syntenic blocks using order and direction between sets of orthologous protein coding genes [75]. Unlike SyMap, this method does not require sequence alignment. The orthologous relationship between genes calculated as described below (see Clusters of orthologous genes section). The Web-interface was used to run the OrthoCluster with default parameters (http://genome.sfu.ca/cgi-bin/orthoclusterdb/runortho.cgi).

### Repeat sequence analysis

Two methods were applied to de novo identify repetitive elements in both raw sequencing data and already assembled scaffolds/contigs. Tedna 1.2.1 [76] was used to assemble transposable element models directly from the repeated fraction of raw Illumina paired-end sequencing reads for each of the genomes. The raw sequencing data for *O. viverrini* (AN: SRR1821044), *C. sinensis* (AN: SRR096372), and *F. hepatica* (AN: ERR576947) were obtained from the Sequence Read Archive database (SRA, NCBI). RepeatModeler 1.0.8 [77] was applied to mine the repeat models from genomic assemblies. The identified repeats from both de novo libraries were automatically annotated using the RepeatClassifier perl script from the RepeatModeler package, which utilizes BLASTN and TBLASTX, against the repeats contained in the RepBase [78] database v. 20,150,807. Tandem and simple repeats, rRNA. and repeat models not affiliated with any superfamily of interspersed repeats represented in RepBase (DNA transposons (DNA), LINE, LTR, Rolling Circle (RC), and SINE) were filtered out and withdrawn from subsequent analyses.

The repeat sequences were clustered by similarity using cd-hit [79] with a clustering threshold of 90%. The produced joint libraries for each of the opisthorchiid species were combined into one opisthorchiid-specific library, which, in turn, was combined with the general RepeatMasker library v. 20,150,807 [77, 78]. The repeats from the generated custom mixed library were mapped on the genome assembly for each of the opisthorchiid genomes using RepeatMasker v. 4.0.5 [77]. The de novo repeat libraries for *F. hepatica* were also merged with the general RepeatMasker library, combined, and used to map the repeats on *F. hepatica* genome assembly only.

The dynamics of transposable elements in opisthorchiid genomes were estimated by taking CpG adjusted Kimura 2P distance to adjust for multiple substitutions of repeats masked by RepeatMasker to their consensus sequences from the library using perl scripts (calcDivergenceFromAlign.pl and createRepeatLandscape.pl) available from the RepeatMasker package [77].

### Gene prediction

The protein-coding genes in the *O. felineus* genome were predicted using a combination of ab initio gene finding algorithms, *O. felineus* transcriptome assemblies, and cross-species alignments of transcript and protein sequences from other trematodes (Additional file 1: Figure S2). Where appropriate, the allowed minimum intron length was set to 20 bp to account for highly occurring microintrons.

For ab initio gene finding, Fgenesh++ [80, 81] and Augustus [82] HMM-based approaches were used. Fgenesh++ included the training on a set of highly homologous cross-species alignments of known proteins, refinement of potential splice sites from alignments of known transcripts, prediction of genes with support from homology to known proteins (NCBI NR database), and ab initio gene prediction in the remaining genome regions. Augustus [82] gene prediction was performed in two ways. Purely ab initio gene prediction was done using the precomputed set of *S. mansoni* parameters. Alternatively, BRAKER1 [83] pipeline was trained against the mapped *O. felineus* RNA-seq data to provide the parameters for further prediction by Augustus. Additionally, core eukaryotic gene model set [84] in *O. felineus* genome was also predicted using CEGMA package.

De novo and genome-guided assemblies of the *O. felineus* transcriptome were built from RNA-seq data using Trinity [85] and cufflinks [86] software. The assembled transcripts were subsequently passed to the PASA [87] comprehensive pipeline to obtain experimentally supported transcript models. Additionally, alignments of proteins from *C. sinensis, O. viverrini,* and *S. mansoni* were constructed from exonerate [88] output.

All resulting prediction (Additional file 2: Table S2) statistics on the predictions of individual gene finding approaches used) were combined into the consensus gene models using EvidenceModeler [87] and further updated with alternatively spliced variants using the iterative runs of PASA pipeline. Transfer RNAs were also predicted by the tRNAscan-SE [89] tool.

In order to explore the properties of transcriptomes in *C. sinensis and O. viverrini,* that may be biased in the available gene annotations (e.g. distribution of intron lengths), we also performed genome-guided transcriptome assembly for these species using cufflinks [86] software.

### Functional annotation of genes and gene sets

The protein-coding genes were annotated by running InterProScan [25] against all natively supported databases (Pfam 29.0, SUPERFAMILY 1.75, SMART 7.1, and PRINTS 42.0; E-value <1E-05, and using the Trinotate pipeline (Additional file 1: Figure S2). Additionally, a homology-based assignment of KEGG pathway identifiers to *O. felineus* protein products was done using KOBAS and KAAS web-services. Gene set enrichment analyses (GSEA) were performed with the use of GOstat [90] R package.

### Trans-splicing analysis

The SL RNA sequence was determined from the overrepresented 5-prime sequences of Trinity de novo assembled transcripts and further verified by overrepresentation of its forward strand in both reads of paired-end RNA-seq libraries as well as by homology to the known flatworm SL RNAs. To determine the sites of trans-splicing in the target transcripts, a fraction of SL RNA containing reads was extracted from RNA-seq data using cutadapt [66], requiring the presence of at least 5 bp of SL sequence. After trimming out SL, the reads were mapped to the reference genome by Tophat v2.1.1 [91]. The splice sites, targeted by SL RNA, that were supported by at least three uniquely mapped reads, were regarded as valid.

**Fig. 9:**
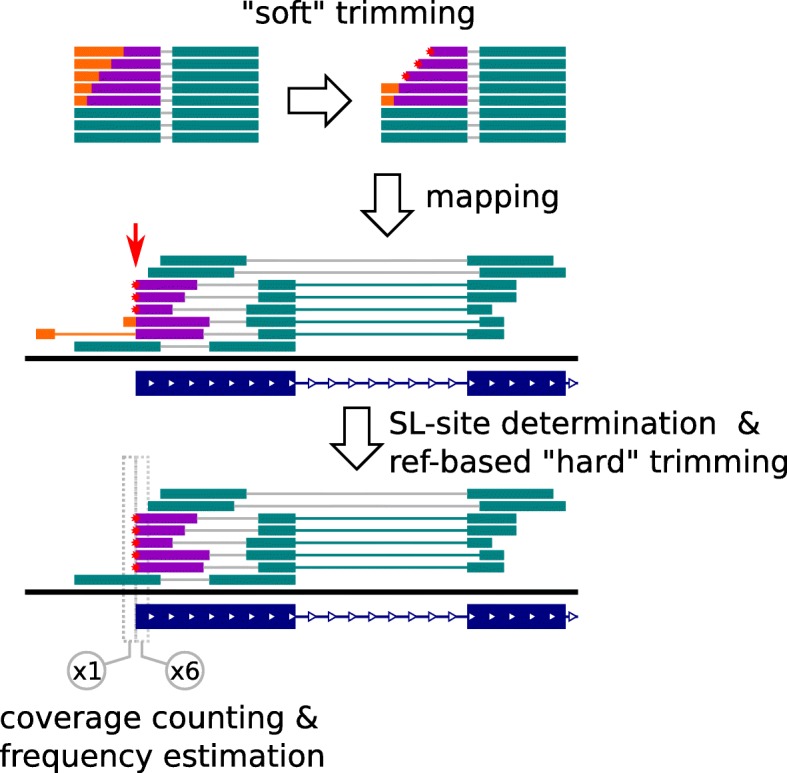
Schematic representation of SLTS sites identification in mapped RNA-seq data. Red arrow indicates SLTS site

To reduce a potential bias in further estimates of splicing efficiency, all RNA-seq reads were subject to the second round of “hard” SL trimming: up to 1-bp match to SL sequence was cut out but only if it was supported by a valid site of SL splicing determined before (Fig. 9). The efficiency (frequency) of SL splicing was defined as 1 minus ratio of read coverage at position upstream of the SL site to that at position downstream of the SL site.

### Interstage and interspecies comparison of gene expression

The available and newly obtained RNA-seq data was preprocessed by cutadapt v1.18 [66] in order to remove low-quality base-calls, adapters and SL-RNA sequences, and aligned using Tophat v2.1.1 [91] with parameters modified to allow the minimum intron size of 20.

The differential expression between *O. felineus* metacercariae and adult worms (three biological replicates of pooled individuals per stage) was analyzed using full *O. felineus* gene annotation and default DESeq2 [92] workflow without any restriction on fold-change and with controlling 5% false-discovery rate by Benjamini–Hochberg (BH) algorithm.

A comparative analysis of gene expression across three opisthorchiid species is complicated by pronounced inconsistencies between their existing gene annotations based on draft genome assemblies. To solve this problem, we identified the gene fragments exactly common for *O. felineus* and two other opisthorchiids. The predicted cDNAs of *O. felineus* were aligned to the *O. viverrini* and *C. sinensis* genomes using Spaln v2.3.0 [93] splice-aware aligner (parameters: -Q7 -Tschimans -yX -yZ2 -yB1 -M1 -LS -S1). Similarly, the best found alignments were mapped back to the *O. felineus* genome. Finally, only reciprocal-best pairs that cover each other by > 90% were retained. *Viverrini* and *C.*

The obtained annotations of ‘orthologous’ coding regions of genes detected for each species were used to prepare gene-wise count tables from RNA-seq data on adult stages. Raw counts were normalized to the actual length of the region in the corresponding species to exclude the bias arising from slight interspecies fluctuations (< 10%) of the latter. RLE (Relative Log Expression, DESeq2) method was used to normalize the data to sequencing depth. DESeq2 [92] R package was utilized to extract the genes with high interspecies differences in expression, since it allowed for taking into account both RNA-seq underdetection bias (e.g. excessively high differences of genes with low read coverage) and intraspecific variation of expression (using the data on three *O. felineus* biological replicates). A threshold-based Wald test was used for statistical testing of more than fourfold difference between the species, with controlling 1% false-discovery rate by BH algorithm.

### Clusters of orthologous genes

The protein sequences of four trematodes, namely, *O. viverrini, C. sinensis, F. hepatica,* and *S. mansoni,* were used to identify orthologous clusters. Proteinortho v. 5.15 [94] with ‘-synteny’ ans ‘-dups = 3’ options set was applied to determine the orthologous clusters among a full set of annotated protein isoforms. *O. felineus*, *F. hepatica* and *S. mansoni* gene models suggested various mRNA splicing variants and, correspondingly, several amino acid sequences for some genes. The produced mRNA-level orthology tables were subsequently merged into gene-level orthologies. This procedure was found to be slightly more sensitive compared to the simple approach of selecting the longest amino acid sequence per gene.

### Phylogeny reconstruction

Phylogenetic trees were constructed based on the amino acid sequences, represented by one sequence for each organism in the clusters of orthologous groups; 1563 groups we used for analysis. Amino acid sequences were aligned in each group of orthologous genes using AQUA v. 1.1 [95] with default parameters. The evolution model was constructed and the alignments were partitioned into uniform blocks with the PartitionFinder2 program [96]. The phylogenetic tree was constructed using MrBayes v. 3.2.6 [97], RAxML v. 8.2.0 [98], and PhyloBayes v. 4.1 [99]. Parameters for MrBayes were set as follows: four MCMC chains 500,000 iterations each, convergence was analyzed by Tracer 1.6 software [100]. RAxML used with 1000 rapid bootstrap inferences and LGF model (selected as best). PhyloBayes using CAT model (options -dgam 6 –cat) was run with two MCMC chains (options -nchain 2 30 0.1100). PhyloBayes results presented on a 50% majority-rule consensus tree calculated with SumTrees v3.3.1 [101].

## Additional files

Additional file 1: **Figure S1.** Genome assembly pipeline. **Figure S2.** Gene annotation pipeline. **Figure S3.** Genome-wide synteny between *O. felineus*, *O. viverrini* and *С. sinensis.* **Figure S4.** Repetitive elements in O. felineus, *C. sinensis*, *O. viverrini* and *F. hepatica* genomes. **Figure S5.** Stage-specific expression of detoxification genes estimated on *O. felineus* transcriptome data. (PDF 438 kb)

Additional file 2: **Table S1.** Sequenced libraries of *O. felineus.* **Table S2.** Functionally annotated genes of *O. felineus.* **Table S3.** *F. hepatica* transposable elements and their classification. **3.2**. *O. felineus* transposable elements and their classification. **3.3**. *O. viverrini* transposable elements and their classification. **3.4**. *C. sinensis* transposable elements and their classification. **Table S4.** Genome-wide synteny between *O. felineus*, *O. viverrini* and *С. sinensis*. **Table S5.** Summary statistics of the gene annotations produced by a number of prediction approaches used for the annotation of *O. felineus* genome assembly. **Table S6.** Differential gene expression in *O. felineus* metacercariae and adult stage. Data are presented as FPKM values (Fragments Per Kilobase Of Exon Per Million Fragments Mapped) with *p*-value adjusted < 0.05. **Table S7.** Orthologous genes detected by ProteinOrtho software with the aid of syntenic information. **Table S8.** Differentially expressed genes in adult *O. felineus* and *O. viverrini* (expression values differing more than fourfold). **Table S9.** Differentially expressed genes in adult *O. felineus* and *C. sinensis* (expression values differing more than fourfold). **Table S10.** Statistics on completeness of genome annotations, as reported by BUSCO software using Eukaryota (EU) & Metazoa (MZ) sets of orthologs. **Table S11.** Evaluation report of *O. felineus* genome assembly produced by REAPR software. (XLSX 4591 kb)

## Abbreviations

ORF: Open reading frame
SF: Supplementary Figs.
SL: Spliced leader
SLTS: Spliced leader trans-splicing
ST: Supplementary tables

## References

[1] Choi BI, Han JK, Hong ST, Lee KH (2004). Clonorchiasis and cholangiocarcinoma: etiologic relationship and imaging diagnosis. Clin Microbiol Rev.

[2] Fried B, Reddy A, Mayer D (2011). Helminths in human carcinogenesis. Cancer Lett.

[3] Pakharukova MY, Mordvinov VA (2016). The liver fluke Opisthorchis felineus: biology, epidemiology and carcinogenic potential. Trans R Soc Trop Med Hyg.

[4] Keiser J, Utzinger J (2005). Emerging foodborne trematodiasis. Emerg Infect Dis.

[5] Food and agriculture Organization of the United Nations/World Health Organization FAO/WHO. “Top ten” list of food-borne parasites released. 2014. http://www.fao.org/news/story/en/item/237323/icode/ Accessed 31 Mar 2018.

[6] Multicriteria-based ranking for risk management of food-borne parasites. Microbiological risk assessment series 23. Food and Agriculture Organization of the United Nations Headquarters. 2014. http://www.waterpathogens.org/book/liver-flukes. Accessed 31 Mar 2018.

[7] Beer SA (2005). Biology of the agent of opisthorchiasis.

[8] Sithithaworn P, Yongvanit P, Duenngai K, Kiatsopit N, Pairojkul C (2014). Roles of liver fluke infection as risk factor for cholangiocarcinoma. J Hepatobiliary Pancreat Sci.

[9] Petney TN, Andrews RH, Saijuntha W, Wenz-Mücke A, Sithithaworn P (2013). The zoonotic, fish-borne liver flukes *Clonorchis sinensis*, *Opisthorchis felineus* and *Opisthorchis viverrini*. Int J Parasitol.

[10] Lun ZR, Gasser RB, Lai DH, Li AX, Zhu XQ, Yu XB (2005). Clonorchiasis: a key foodborne zoonosis in China. Lancet Infect Dis.

[11] Brusentsov II, Katokhin AV, Brusentsova IV, Shekhovtsov SV, Borovikov SN, Goncharenko GG (2013). Low genetic diversity in wide-spread Eurasian liver fluke *Opisthorchis felineus* suggests special demographic history of this trematode species. PLoS One.

[12] Sun J, Huang Y, Huang H, Liang P, Wang X, Mao Q (2013). Low divergence of *Clonorchis sinensis* in China based on multilocus analysis. PLoS One.

[13] Laoprom N, Sithithaworn P, Andrews RH, Ando K, Laha T, Klinbunga S (2012). Population genetic structuring in *Opisthorchis viverrini* over various spatial scales in Thailand and Lao PDR. PLoS Negl Trop Dis.

[14] Zadesenets KS, Katokhin AV, Mordvinov VA, Rubtsov NB (2012). Comparative cytogenetics of opisthorchid species (Trematoda, Opisthorchiidae). Parasitol Int.

[15] Wang X, Chen W, Huang Y, Sun J, Men J, Liu H (2011). The draft genome of the carcinogenic human liver fluke *Clonorchis sinensis*. Genome Biol.

[16] Young ND, Nagarajan N, Lin SJ, Korhonen PK, Jex AR, Hall RS (2014). The *Opisthorchis viverrini* genome provides insights into life in the bile duct. Nat Commun.

[17] Huang Y, Chen W, Wang X, Liu H, Chen Y, Guo L (2013). The carcinogenic liver fluke, *Clonorchis sinensis*: new assembly, reannotation and analysis of the genome and characterization of tissue transcriptomes. PLoS One.

[18] Pomaznoy MY, Logacheva MD, Young ND, Penin AA, Ershov NI, Katokhin AV (2016). Whole transcriptome profiling of adult and infective stages of the trematode *Opisthorchis felineus*. Parasitol Int.

[19] Cwiklinski K, Dalton JP, Dufresne PJ, La Course J, Williams DJ, Hodgkinson J (2015). The *Fasciola hepatica* genome: gene duplication and polymorphism reveals adaptation to the host environment and the capacity for rapid evolution. Genome Biol.

[20] Chalopin D, Naville M, Plard F, Galiana D, Volff JN (2015). Comparative analysis of transposable elements highlights mobilome diversity and evolution in vertebrates. Genome Biol Evol..

[21] Soderlund C, Bomhoff M, Nelson W (2011). SyMAP v3.4: a turnkey synteny system with application to plant genomes. Nucleic Acids Res.

[22] Allen MA, Hillier LW, Waterston RH, Blumenthal T (2011). A global analysis of C. elegans trans-splicing. Genome Res.

[23] Lei Q, Li C, Zuo Z, Huang C, Cheng H, Zhou R (2016). Evolutionary insights into RNA trans-splicing in vertebrates. Genome Biol Evol..

[24] Protasio AV, Tsai IJ, Babbage A, Nichol S, Hunt M, Aslett MA (2012). A systematically improved high quality genome and transcriptome of the human blood fluke *Schistosoma mansoni*. PLoS Negl Trop Dis.

[25] Jones P, Binns D, Chang HY, Fraser M, Li W, McAnulla C (2014). InterProScan 5: genome-scale protein function classification. Bioinformatics..

[26] Marchler-Bauer A, Bo Y, Han L, He J, Lanczycki CJ, Lu S (2017). CDD/SPARCLE: functional classification of proteins via subfamily domain architectures. Nucleic Acids Res.

[27] Sigrist CJA, de Castro E, Cerutti L, Cuche BA, Hulo N, Bridge A, Bougueleret L, Xenarios I (2013). New and continuing developments at PROSITE. Nucleic Acids Res.

[28] Cuesta-Astroz Y, Oliveira FS, Nahum LA, Oliveira G (2017). Helminth secretomes reflect different lifestyles and parasitized hosts. Int J Parasitol.

[29] Matouskova P, Vokřál I, Lamka J, Skálová L (2016). The role of xenobiotic-metabolizing enzymes in anthelmintic deactivation and resistance in helminths. Trends Parasitol.

[30] Pakharukova MY, Vavilin VA, Sripa B, Laha T, Brindley PJ, Mordvinov VA (2015). Functional analysis of the unique cytochrome P450 of the liver fluke *Opisthorchis felineus*. PLoS Negl Trop Dis.

[31] Ziniel PD, Karumudi B, Barnard AH, Fisher EM, Thatcher GR, Podust LM (2015). The *Schistosoma mansoni* cytochrome P450 (CYP3050A1) is essential for worm survival and egg development. PLoS Negl Trop Dis.

[32] Mordvinov VA, Shilov AG, Pakharukova MY (2017). Anthelmintic activity of cytochrome P450 inhibitors miconazole and clotrimazole: in-vitro effect on the liver fluke *Opisthorchis felineus*. Int J Antimicrob Agents.

[33] Gouveia MJ, Pakharukova MY, Laha T, Sripa B, Maksimova GA, Rinaldi G (2017). Infection with *Opisthorchis felineus* induces intraepithelial neoplasia of the biliary tract in a rodent model. Carcinogenesis..

[34] Gouveia MJ, Santos J, Brindley PJ, Rinaldi G, Lopes C, Santos LL (2015). Estrogen-like metabolites and DNA-adducts in urogenital schistosomiasis-associated bladder cancer. Cancer Lett.

[35] Pakharukova MY, Ershov NI, Vorontsova EV, Katokhin AV, Merkulova TI, Mordvinov VA (2012). Cytochrome P450 in fluke *Opisthorchis felineus*: identification and characterization. Mol Biochem Parasitol.

[36] Laing R, Kikuchi T, Martinelli A, Tsai IJ, Beech RN, Redman E (2013). The genome and transcriptome of *Haemonchus contortus*, a key model parasite for drug and vaccine discovery. Genome Biol.

[37] Lindblom TH, Dodd AK (2006). Xenobiotic detoxification in the nematode Caenorhabditis elegans. J Exp Zool.

[38] Saier MH, Reddy VS, Tamang DG, Västermark A (2014). The transporter classification database. Nucleic Acids Res.

[39] Wong K, Ma J, Rothnie A, Biggin PC, Kerr ID (2014). Towards understanding promiscuity in multidrug efflux pumps. Trends Biochem Sci.

[40] Mordvinov VA, Ershov NI, Pirozhkova DS, Pakharukov YV, Pakharukova MY (2017). ABC transporters in the liver fluke *Opisthorchis felineus*. Mol Biochem Parasitol.

[41] Pelosi Paolo, Zhu Jiao, Knoll Wolfgang (2018). Odorant-Binding Proteins as Sensing Elements for Odour Monitoring. Sensors.

[42] Smout MJ, Sotillo J, Laha T, Papatpremsiri A, Rinaldi G, Pimenta RN (2015). Carcinogenic parasite secretes growth factor that accelerates wound healing and potentially promotes neoplasia. PLoS Pathog.

[43] Pila EA, Gordy MA, Phillips VK, Kabore AL, Rudko SP, Hanington PC (2016). Endogenous growth factor stimulation of hemocyte proliferation induces resistance to Schistosoma mansoni challenge in the snail host. Proc Natl Acad Sci U S A.

[44] Zadesenets KS, Karamysheva TV, Katokhin AV, Mordvinov VA, Rubtsov NB (2012). Distribution of repetitive DNA sequences in chromosomes of five opisthorchid species (Trematoda, Opisthorchiidae). Parasitol Int.

[45] Pakharukova MY, Shilov AG, Pirozhkova DS, Katokhin AV, Mordvinov VA (2015). The first comprehensive study of praziquantel effects *in vivo* and *in vitro* on European liver fluke *Opisthorchis felineus* (Trematoda). Int J Antimicrob Agents.

[46] Mordvinov VA, Furman DP (2010). The Digenea parasite *Opisthorchis felineus*: a target for the discovery and development of novel drugs. Infect Disord Drug Targets.

[47] Paland S, Lynch M (2006). Transitions to asexuality result in excess amino acid substitutions. Science..

[48] Petney TN, Andrews RH, Saijuntha W, Tesana S, Prasopdee S, Kiatsopit N, et al. Taxonomy, ecology and population genetics of *Opisthorchis viverrini* and its intermediate hosts. Adv Parasitol. 2018;101:1–39. 10.1016/bs.apar.2018.05.001 29907251

[49] Kang S, Sultana T, Loktev VB, Wongratanacheewin S, Sohn WM, Eom KS (2008). Molecular identification and phylogenetic analysis of nuclear rDNA sequences among three opisthorchid liver fluke species (Opisthorchiidae: Trematoda). Parasitol Int.

[50] Katokhin AV, Shekhovtsov SV, Konkow S, Yurlova NI, Serbina EA, Vodianitskai SN (2008). Assessment of the genetic distinctions of Opisthorchis felineus from O. viverrini and Clonorchis sinensis by ITS2 and CO1 sequences. Dokl Biochem Biophys.

[51] Saijuntha W, Sithithaworn P, Wongkham S, Laha T, Chilton NB, Petney TN (2008). Mitochondrial DNA sequence variation among geographical isolates of Opisthorchis viverrini in Thailand and Lao PDR, and phylogenetic relationships with other trematodes. Parasitology.

[52] Pitaksakulrat O, Webster BL, Webster JP, Laha T, Saijuntha W, Lamberton PH (2018). Phylogenetic relationships within the Opisthorchis viverrini species complex with specific analysis of O. viverrini sensu lato from Sakon Nakhon, Thailand by mitochondrial and nuclear DNA sequencing. Infect Genet Evol.

[53] Wang D, Young ND, Koehler AV, Tan P, Sohn WM, Korhonen PK, Gasser RB (2017). Mitochondrial genomic comparison of Clonorchis sinensis from South Korea with other isolates of this species. Infect Genet Evol.

[54] Liu GH, Gasser RB, Young ND, Song HQ, Ai L, Zhu XQ (2014). Complete mitochondrial genomes of the ‘intermediate form’of Fasciola and Fasciola gigantica, and their comparison with F. hepatica. Parasit Vectors.

[55] Shekhovtsov SV, Katokhin AV, Romanov KV, Besprozvannykh VV, Fedorov KP, Yurlova NI (2009). A novel nuclear marker, pm-int9, for phylogenetic studies of Opisthorchis felineus, Opisthorchis viverrini, and Clonorchis sinensis (Opisthorchiidae, Trematoda). Parasitol Res.

[56] Cai XQ, Liu GH, Song HQ, Wu CY, Zou FC, Yan HK (2012). Sequences and gene organization of the mitochondrial genomes of the liver flukes Opisthorchis viverrini and Clonorchis sinensis (Trematoda). Parasitol Res.

[57] Yu J, Yang Z, Kibukawa M, Paddock M, Passey DA, Wong GK (2002). Minimal introns are not "junk". Genome Res.

[58] Bondarenko VS, Gelfand MS (2016). Evolution of the exon-intron structure in ciliate genomes. PLoS One.

[59] Tsai IJ, Zarowiecki M, Holroyd N, Garciarrubio A, Sánchez-Flores A, Brooks KL (2013). The genomes of four tapeworm species reveal adaptations to parasitism. Nature..

[60] Hahn C, Fromm B, Bachmann L (2014). Comparative genomics of flatworms (Platyhelminthes) reveals shared genomic features of ecto- and endoparastic neodermata. Genome Biol Evol.

[61] Maksimova GA, Pakharukova MY, Kashina EV, Zhukova NA, Kovner AV, Lvova MN (2017). Effect of *Opisthorchis felineus* infection and dimethylnitrosamine administration on the induction of cholangiocarcinoma in Syrian hamsters. Parasitol Int.

[62] WormBase ParaSite. WBPS6 release. https://parasite.wormbase.org/index.html. Accessed 31 Mar 2016.

[63] Howe KL, Bolt BJ, Shafie M, Kersey P, Berriman M (2017). WormBase ParaSite – a comprehensive resource for helminth genomics. Mol Biochem Parasitol.

[64] Berriman M, Haas BJ, LoVerde PT, Wilson RA, Dillon GP, Cerqueira GC (2009). The genome of the blood fluke *Schistosoma mansoni*. Nature..

[65] Wasik K, Gurtowski J, Zhou X, Ramos OM, Delás MJ, Battistoni G (2015). Genome and transcriptome of the regeneration-competent flatworm, *Macrostomum lignano*. Proc Natl Acad Sci U S A.

[66] Martin M (2011). Cutadapt removes adapter sequences from high-throughput sequencing reads. EMBnet J.

[67] Zhang J, Kobert K, Flouri T, Stamatakis A (2014). PEAR: a fast and accurate Illumina paired-end reAd mergeR. Bioinformatics..

[68] Gnerre S, Maccallum I, Przybylski D, Ribeiro FJ, Burton JN, Walker BJ (2011). High-quality draft assemblies of mammalian genomes from massively parallel sequence data. Proc Natl Acad Sci U S A.

[69] Sahlin K, Vezzi F, Nystedt B, Lundeberg J, Arvestad L (2014). BESST-efficient scaffolding of large fragmented assemblies. BMC Bioinformatics..

[70] Li R, Fan W, Tian G, Zhu H, He L, Cai J (2010). The sequence and de novo assembly of the giant panda genome. Nature..

[71] Swain MT, Tsai IJ, Assefa SA, Newbold C, Berriman M, Otto TD (2012). A post-assembly genome-improvement toolkit (PAGIT) to obtain annotated genomes from contigs. Nat Protoc.

[72] Hunt M, Kikuchi T, Sanders M, Newbold C, Berriman M, Otto TD (2013). REAPR: a universal tool for genome assembly evaluation. Genome Biol.

[73] Simão FA, Waterhouse RM, Ioannidis P, Kriventseva EV, Zdobnov EM (2015). BUSCO: assessing genome assembly and annotation completeness with single-copy orthologs. Bioinformatics..

[74] Kurtz S, Phillippy A, Delcher AL, Smoot M, Shumway M, Antonescu C (2004). Versatile and open software for comparing large genomes. Genome Biol.

[75] Ng MP, Vergara IA, Frech C, Chen Q, Zeng X, Pei J (2009). OrthoClusterDB: an online platform for synteny blocks. BMC bioinformatics.

[76] Zytnicki M, Akhunov E, Quesneville H (2014). Tedna: a transposable element de novo assembler. Bioinformatics..

[77] Smit AFA, Hubley R. RepeatModeler Open-1.0. 2008-2015. 2015. http://www.repeatmasker.org. Accessed 31 Mar 2018.

[78] Jurka J, Kapitonov VV, Pavlicek A, Klonowski P, Kohany O, Walichiewicz J (2005). Repbase update, a database of eukaryotic repetitive elements. Cytogenet Genome Res.

[79] Fu L, Niu B, Zhu Z, Wu S, Li W (2012). CD-HIT: accelerated for clustering the next-generation sequencing data. Bioinformatics..

[80] Salamov A, Solovyev V (2000). Ab initio gene finding in Drosophila genomic DNA. Genome Res.

[81] Solovyev Victor, Kosarev Peter, Seledsov Igor, Vorobyev Denis (2006). Genome Biology.

[82] Stanke M, Morgenstern B (2005). AUGUSTUS: a web server for gene prediction in eukaryotes that allows user-defined constraints. Nucleic Acids Res.

[83] Hoff KJ, Lange S, Lomsadze A, Borodovsky M, Stanke M (2016). BRAKER1: unsupervised RNA-Seq-based genome annotation with GeneMark-ET and AUGUSTUS. Bioinformatics..

[84] Parra G, Bradnam K, Korf I (2007). CEGMA: a pipeline to accurately annotate core genes in eukaryotic genomes. Bioinformatics..

[85] Haas BJ, Papanicolaou A, Yassour M, Grabherr M, Blood PD (2013). Bowden, et al. De novo transcript sequence reconstruction from RNA-Seq: reference generation and analysis with trinity. Nat Protoc.

[86] Pollier J, Rombauts S, Goossens A (2013). Analysis of RNA-Seq data with TopHat and cufflinks for genome-wide expression analysis of jasmonate-treated plants and plant cultures. Methods Mol Biol.

[87] Haas BJ, Salzberg SL, Zhu W, Pertea M, Allen JE, Orvis J (2008). Automated eukaryotic gene structure annotation using EVidenceModeler and the program to assemble spliced alignments. Genome Biol.

[88] Slater GS, Birney E (2005). Automated generation of heuristics for biological sequence comparison. BMC Bioinformatics.

[89] Lowe TM, Eddy SR (1997). tRNAscan-SE: a program for improved detection of transfer RNA genes in genomic sequence. Nucleic Acids Res.

[90] Beissbarth T, Speed TP (2004). GOstat: find statistically overrepresented gene ontologies within a group of genes. Bioinformatics..

[91] Kim D, Pertea G, Trapnell C, Pimentel H, Kelley R, Salzberg SL (2013). TopHat2: accurate alignment of transcriptomes in the presence of insertions, deletions and gene fusions. Genome Biol.

[92] Love MI, Huber W, Anders S (2014). Moderated estimation of fold change and dispersion for RNA-seq data with DESeq2. Genome Biol.

[93] Iwata H, Gotoh O (2012). Benchmarking spliced alignment programs including Spaln2, an extended version of Spaln that incorporates additional species-specific features. Nucleic Acids Res.

[94] Lechner M, Findeiß S, Steiner L, Marz M, Stadler PF, Prohaska SJ (2011). Proteinortho: detection of (co-) orthologs in large-scale analysis. BMC bioinformatics.

[95] Muller J, Creevey CJ, Thompson JD, Arendt D, Bork P (2009). AQUA: automated quality improvement for multiple sequence alignments. Bioinformatics.

[96] Lanfear R, Frandsen PB, Wright AM, Senfeld T, Calcott B (2017). PartitionFinder 2: new methods for selecting partitioned models of evolution for molecular and morphological phylogenetic analyses. Mol Biol Evol.

[97] Ronquist F, Teslenko M, Van Der Mark P, Ayres DL, Darling A, Höhna S (2012). MrBayes 3.2: efficient Bayesian phylogenetic inference and model choice across a large model space. Syst Biol.

[98] Stamatakis A (2014). RAxML version 8: a tool for phylogenetic analysis and post-analysis of large phylogenies. Bioinformatics..

[99] Lartillot N, Rodrigue N, Stubbs D, Richer J (2013). PhyloBayes MPI: phylogenetic reconstruction with infinite mixtures of profiles in a parallel environment. Syst Biol.

[100] Rambaut A, Drummond AJ, Xie D, Baele G, Suchard MA (2018). Posterior summarization in Bayesian phylogenetics using tracer 1.7. Syst Biol.

[101] Sukumaran J, Holder MT (2010). DendroPy: a Python library for phylogenetic computing. Bioinformatics..

