# Supplementary material for: New insights from Opisthorchis felineus genome: update on genomics of the epidemiologically important liver flukes
Source: BMC Genomics. 2019 May 22;20:399. doi: 10.1186/s12864-019-5752-8 (PMC6530080; doi:10.1186/s12864-019-5752-8)
Supplement: Supplementary file 1 — Figure S1. Genome assembly pipeline. Figure S2. Gene annotation pipeline. Figure S3. Genome-wide synteny between O. felineus, O. viverrini and С. sinensis. Figure S4. Repetitive elements in O. felineus, C. sinensis, O. viverrini and F. hepatica genomes. Figure S5. Stage-specific expression of detoxification genes estimated on O. felineus transcriptome data. (PDF 438 kb) [file 12864_2019_5752_MOESM1_ESM.pdf]

Supplementary materials: figures

SF1. Genome assembly pipeline

SF2. Gene annotation pipeline

SF3. Genome-wide synteny between O. felineus, O. viverrini and C. sinensis

SF4. Repetitive elements in O. felineus, C. sinensis, O. viverrini and F. hepatica genomes

SF5. Stage-specific expression of detoxification genes estimated on O. felineus transcriptome data.

SF1. Genome assembly pipeline

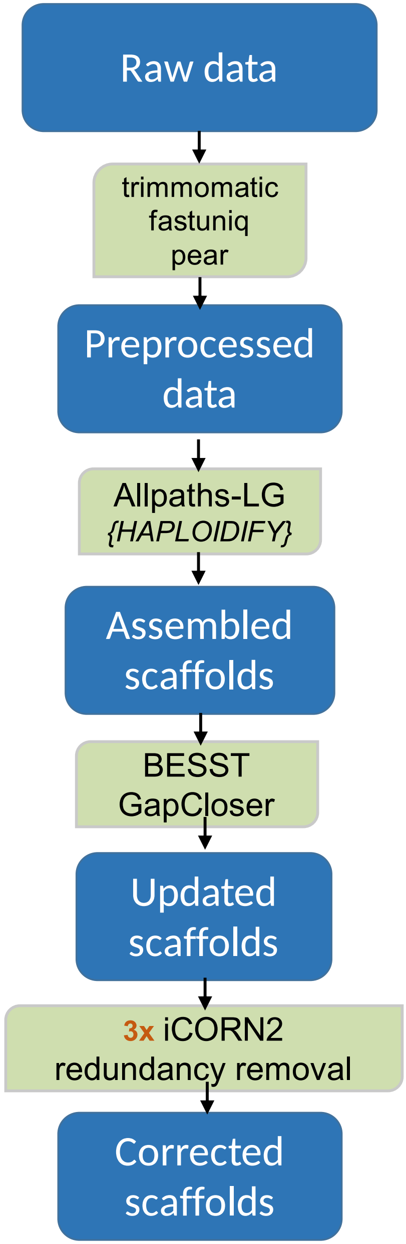

SF2. Gene annotation pipeline

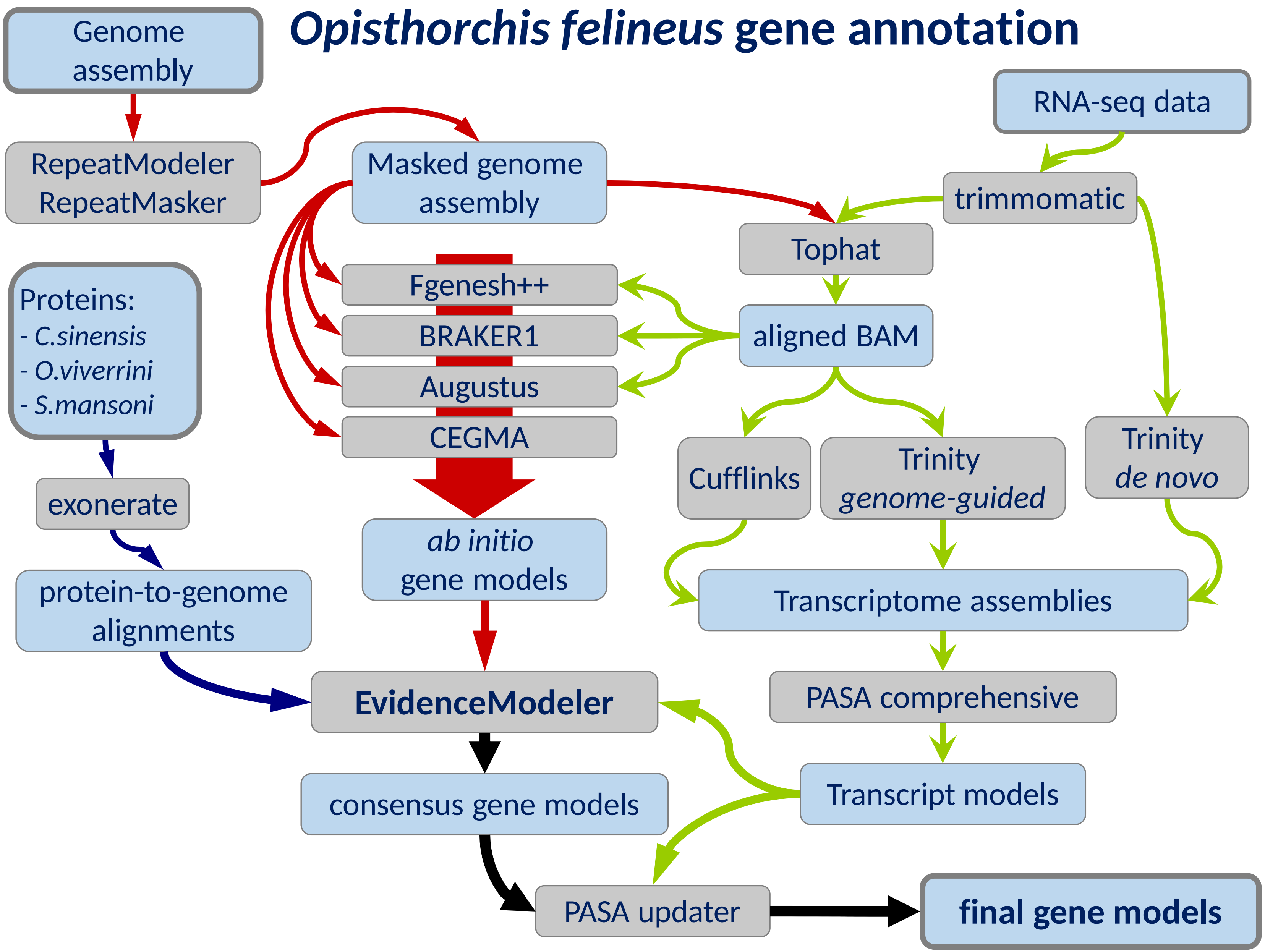

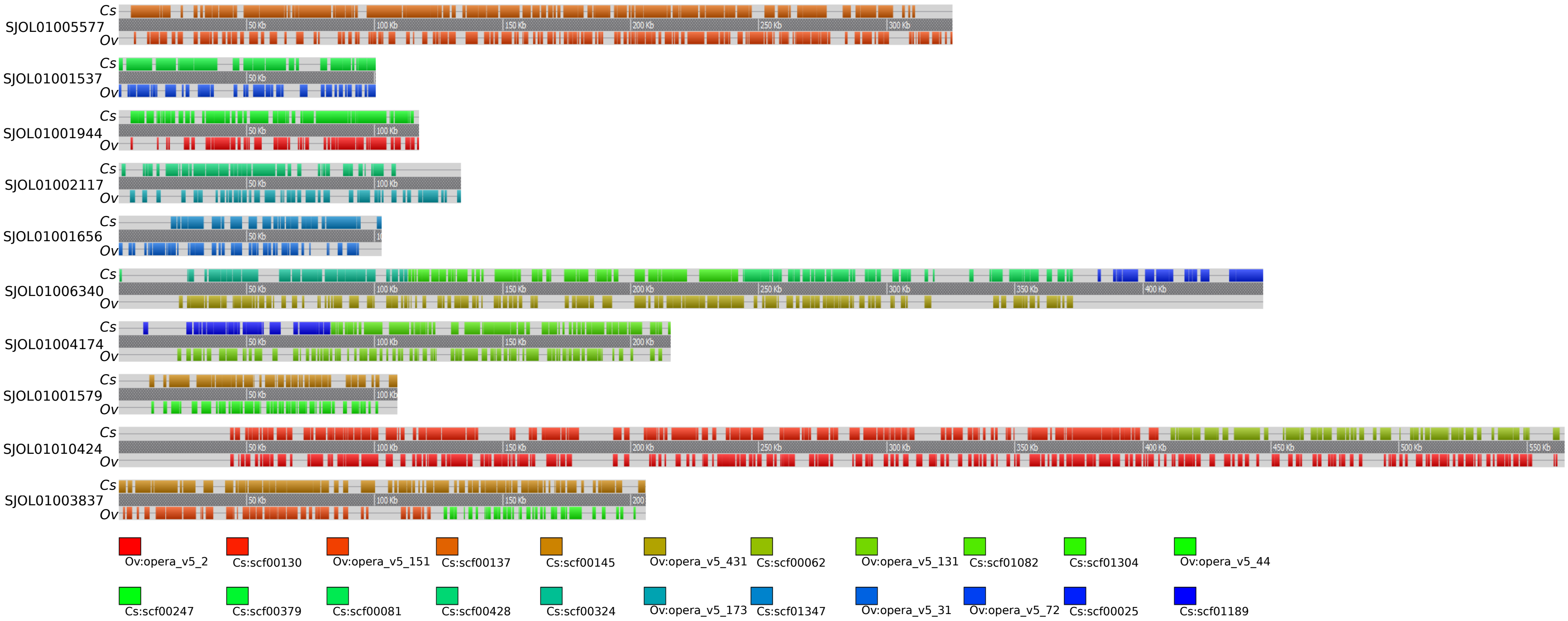

SF3. Alignment of the top 10 *O. felineus* contigs with the highest coverage by *O. viverrini* and *C. sinensis* genomic sequences. The *O. felineus* contig sequence is represented by middle grey bar. Alignment of the *C. sinensis* genomic sequences is shown by upper bar denoted by ‘*Cs*' on the left. Alignment of the *O. viverrini* genomic sequences is shown by upper bar denoted by ‘*Ov*' on the left. Aligned sequences from the same contig have the same color. Colors of *O. viverrini* and *C. sinensis* contigs are sown as color bars at the bottom of the figure. Colors reflects relative degree of contigs similarity from highest (red) to lowest (blue).

SF4.Repetitive elements in *O. felineus*, *C. sinensis*, *O. viverrini*, and *F. hepatica* genomes

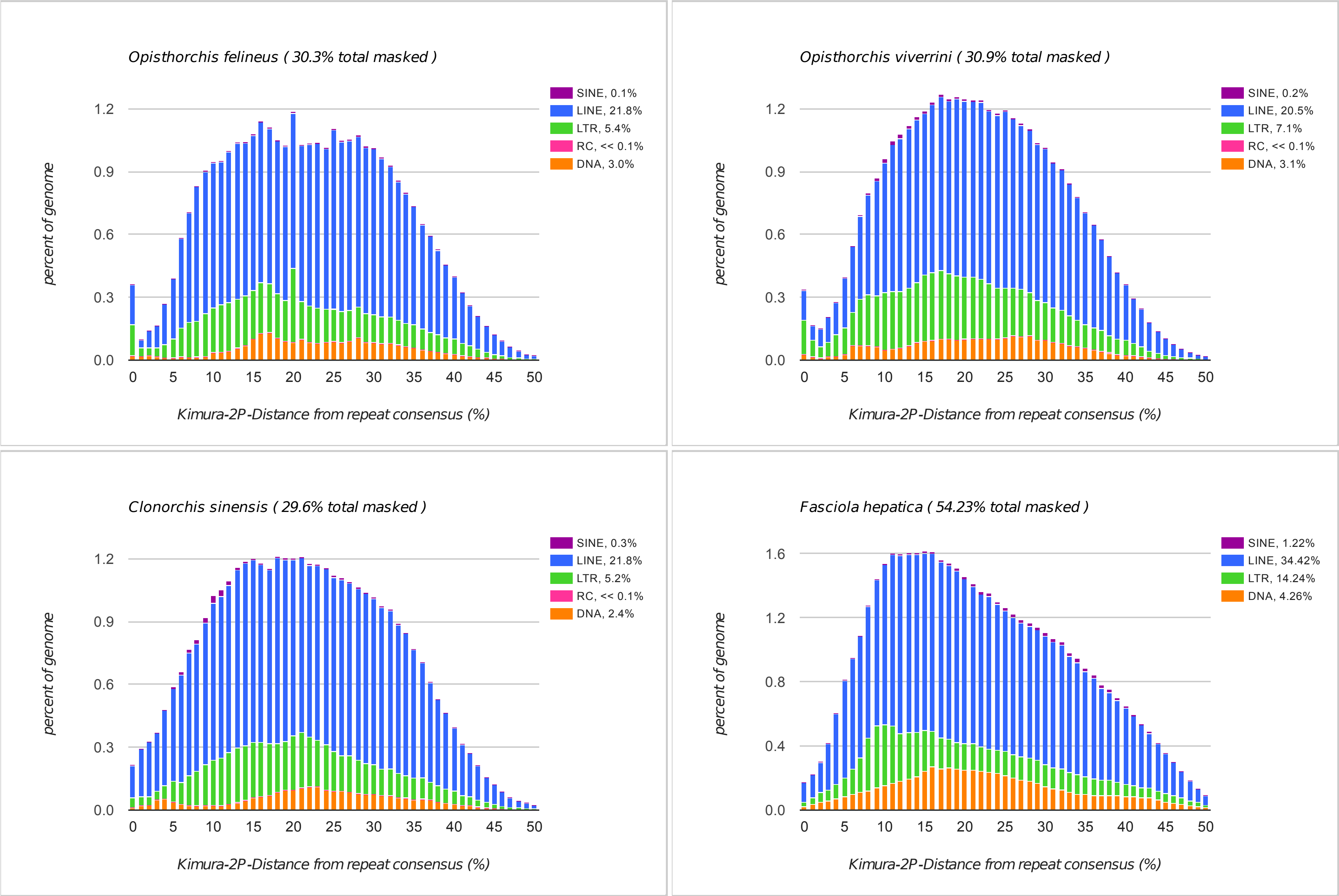

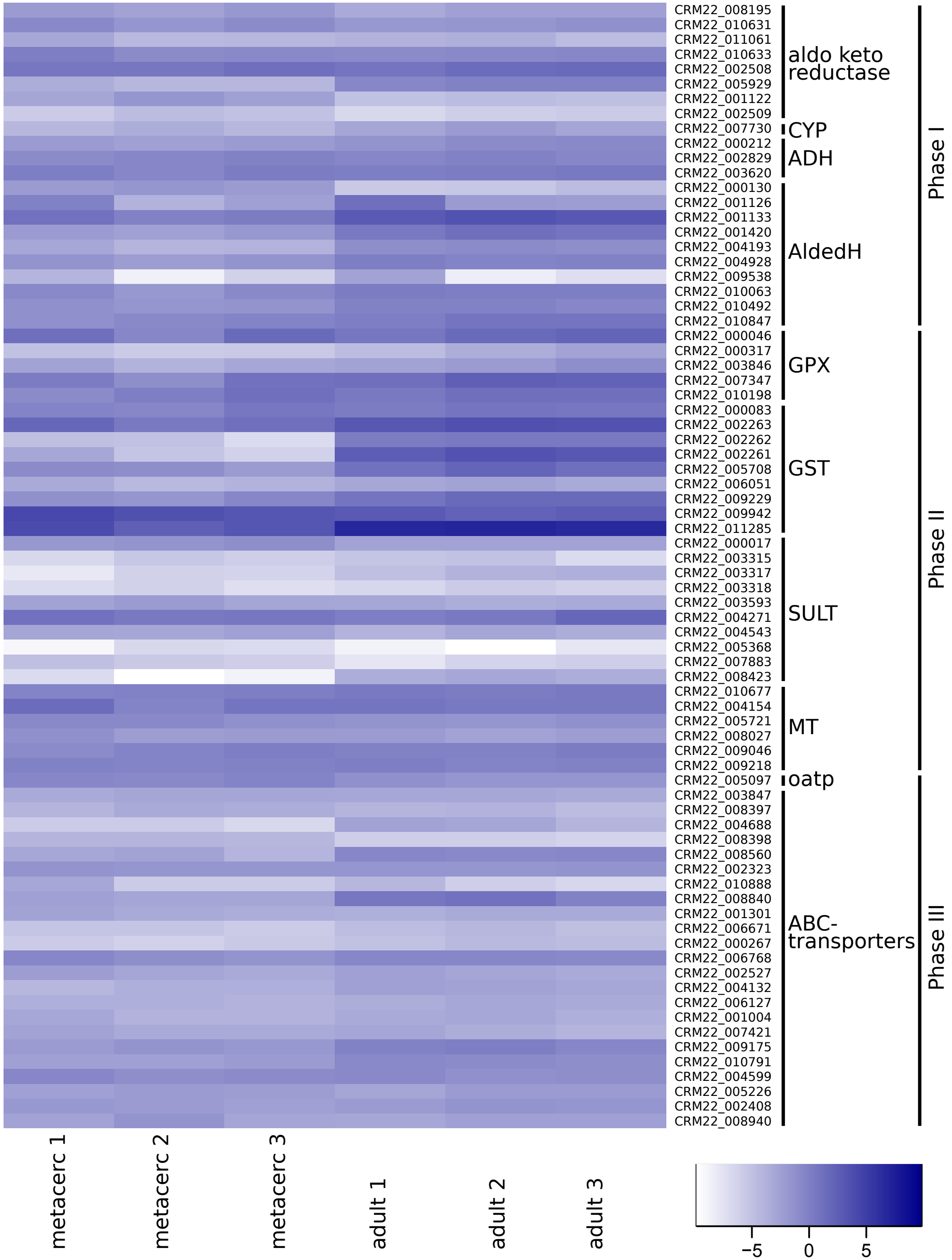

SF5. Stage-specific expression of detoxification genes estimated on *O. felineus* transcriptome data. To analyze expression differences between genes, normalized read counts from RNA-seq data were transformed into $\log_2$(FPKM) values, i.e. transcript abundance.
